# How much, if anything, do we know about sperm chromosomes of Robertsonian translocation carriers?

**DOI:** 10.1007/s00018-020-03560-5

**Published:** 2020-06-08

**Authors:** Ewa Wiland, Marta Olszewska, Tomasz Woźniak, Maciej Kurpisz

**Affiliations:** Institute of Human Genetics, Polish Academy of Sciences, ul. Strzeszynska 32, 60-479 Poznan, Poland

**Keywords:** Rare and common robertsonian translocations, Sperm chromosomes

## Abstract

In men with oligozoospermia, Robertsonian translocations (RobTs) are the most common type of autosomal aberrations. The most commonly occurring types are rob(13;14) and rob(14;21), and other types of RobTs are described as ‘rare’ cases. Based on molecular research, all RobTs can be broadly classified into Class 1 and Class 2. Class 1 translocations produce the same breakpoints within their RobT type, but Class 2 translocations are predicted to form during meiosis or mitosis through a variety of mechanisms, resulting in variation in the breakpoint locations. This review seeks to analyse the available data addressing the question of whether the molecular classification of RobTs into Classes 1 and 2 and/or the type of DD/GG/DG symmetry of the involved chromosomes is reflected in the efficiency of spermatogenesis. The lowest frequency value calculated for the rate of alternate segregants was found for rob(13;15) carriers (Class 2, symmetry DD) and the highest for rob(13;21) carriers (Class 2, DG symmetry). The aneuploidy values for the rare RobT (Class 2) and common rob(14;21) (Class 1) groups together exhibited similarities while differing from those for the common rob(13;14) (Class 1) group. Considering the division of RobT carriers into those with normozoospermia and those with oligoasthenozoospermia, it was found that the number of carriers with elevated levels of aneuploidy was unexpectedly quite similar and high (approx. 70%) in the two subgroups. The reason(s) that the same RobT does not always show a similar destructive effect on fertility was also pointed out.

**Electronic supplementary material:**

The online version of this article (10.1007/s00018-020-03560-5) contains supplementary material, which is available to authorized users.

## Introduction

Robertsonian translocation (RobT) is the central fusion of the long arms of two acrocentric chromosomes. In humans, five pairs of acrocentric chromosomes (13, 14, 15, 21, and 22) can form ten different nonhomologous RobTs: rob(13;14)(q10;q10), rob(13;15)(q10;q10), rob(13;21)(q10;q10), rob(13;22)(q10;q10), rob(14;15)(q10;q10), rob(14;21)(q10;q10), rob(14;22)(q10;q10), rob(15;21)(q10;q10), rob(15;22)(q10;q10), and rob(21;22)(q10;q10). The most commonly occurring ones are rob(13;14), observed in approximately 73–85% of all RobTs**,** followed by rob(14;21), found in 10% of cases [1, 2]. Other particular types of RobT are described as ‘rare’ (estimated rob(13;21) approximately 2%; rob(13;22) approximately 1% and rob(15;22) approximately 0.6% of all RobT) [1, 3, 4]. As a consequence of the central fusion, RobT carriers exhibit 45 chromosomes, making them easily identifiable. RobTs are the type of translocation that is simplest to recognize; therefore, it is not surprising that it was the first chromosomal translocation in humans to be described by the Lejeune group, in 1965 [5]. Lejeune’s group reported a karyotype of 45 chromosomes, and they interpreted the fusion involved as being composed of 22q and either 14q or 15q [quote from [6]]. The name of this kind of aberration originates from the name of the American biologist W.R.B. Robertson, who first described this type of translocation in 1916 in grasshoppers [7]. Nonhomologous RobTs are common in humans, with an incidence of approximately 1 in 1000–1230 births (0.1–0.123%) [8–13]. Such a high frequency indicates that RobTs do not occur in the population by coincidence. RobT can either be inherited from a carrier parent or appear de novo (in ~ 50% of cases) [14, 15].

Homozygosity for RobT has been reported very rarely, usually for the most common RobTs, rob(13;14) and rob(14;21). Most reported cases occur in phenotypically normal individuals and arise from inbreeding within a family that carries a familial RobT. In addition, most carriers are fertile, and as expected, their offspring are heterozygous for the RobT [16, 17].

The mechanisms of balanced heterologous RobT formation have not yet been identified, but likely reflect the dynamic nature of the acrocentric short-arm chromatin and the tendency for these regions to participate in exchanges. All ten short arms of acrocentric chromosomes exhibit a specific genomic organization, and they share several highly similar blocks of repetitive DNA, including the following satellite sequences: the p11 region of these chromosomes includes satellite DNAs I, II, III, IV, and β; the p12 region, referred to as “the stalks,” contains multiple copies of the genes coding for the 18S and 28S ribosomal RNA (nucleolar organizer region); and the p13 region terminates with β-satellite DNA and telomeric sequences [18–21]. Acrocentric fusions have been proposed to occur via incomplete homologous or nonhomologous recombination between short-arm repeats or through the repair of short-arm DNA damage, which is corrected by a similar short-arm DNA sequence on a nearby nonhomologous acrocentric chromosome [15, 18–21]. In most cases, the regions of the breaks are located just beyond the centromere. A singleton chromosome with two centromeres is then formed (a dicentric chromosome), one of which loses the structure of the centromere and remains inactive. The simultaneously created fragment without a centromere (acentric) is lost during subsequent cell divisions [22, 23]. There are also two possible rare mechanisms of RobT formation that produce a chromosome translocation, resulting in a chromosome with one centromere (monocentric): fusion at the centromere (centric fusion) or union following breakage of one short arm and one long arm (essentially, a whole-arm reciprocal translocation) [24]. Moreover, it was shown that the presence of an RobT does not have a considerable effect on smaller structural aberrations (e.g., breaks, gaps, and deletions) [25]. In most balanced cases, no phenotypic significance is linked to the loss of the short chromosome arms, probably because these arms contain only nucleolus-organizing regions (NORs), which exist as clusters of approximately 400 copies of 43 kb ribosomal DNA (rDNA) and are located between centromeric and telomeric heterochromatin [26, 27]. However, there are reports, suggesting that RobT carriers might be at greater risk of haematological disorders and breast cancer [28–32].

Based on molecular research, it has been suggested that all heterologous RobTs can be broadly classified into Class 1 and Class 2 [15, 20]. Class 1 RobTs occur nonrandomly in the population; most of them (∼95%) occur during oogenesis. The remaining part of reported cases was postzygotically formed between a maternal and a paternal chromosome or were paternally derived [20, 15]. Their breakpoints are in ~ 98% consistent locations which suggest a distinct reproducible mechanism of formation. The proposed mechanism is recombination between homologous sequences that are shared on the short arms of the acrocentric chromosomes. This would lead to specific, recurrent breakpoints and may account for the frequent mutation events observed in common translocations. It has been proposed that the homologous sequences on the short arms of chromosomes 13 and 21 are arranged in the opposite orientation on chromosome 14. Class 1 includes the vast majority of cases of common rob(13;14) and all cases of the common rob(14;21). Specifically, examined all de novo rob(14;21) have similar breakpoints located within highly repetitive satellite III DNA sequences and between a satellite I subfamily pTRI-6 and the rRNA genes on chromosome 21 [14, 20]. Class 1 may also include some rare RobTs that involve chromosome 14 (e.g., rob(14;22)) whose the breakpoints on chromosome 14 fall between satellite subfamilies pTRS-47 and pTRS-63 [20]. In contrast, Class 2 comprises the majority of rare RobTs. These cases can be formed during meiosis or mitosis and have variable parental origins; during oogenesis in ~ 70%. Although both classes of RobTs occur predominantly during meiosis and repair of DNA is the cause of the translocation, Class 2 RobTs are predicted to have varied breakpoints and probably arise through a more “random” mechanism or a variety of mechanisms (for example, nonhomologous end joining and illegitimate recombination, or homologous recombination in hypervariable minisatellite DNA). Within Class 2, rob(14;15) was found to exhibit the most variable breakpoint locations and timing of formation [33]. Class 2 may also include some common RobTs that have different breakpoint locations or post-zygotic formation [15, 19, 20, 33].

Undoubtedly, it would be advisable to perform a further high-resolution analysis of larger groups of each type of RobTs to figure out whether the molecular classification into Class 1 and Class 2 is fully justified.

As mentioned above, for the majority of people with heterologous RobT, carrying the translocation in its balanced form does not result in phenotypic consequences, with the important exceptions of increased risk of miscarriage and reduced fertility [34–37]. These consequences result from the fact that during meiosis, the rearranged acrocentric chromosomes in the pachytene stage form a configuration composed of three chromosomes (trivalent) as a result of paired homologous fragments. In anaphase, these three chromosomes segregate to the gametes, resulting in the following segregation types: alternate, adjacent 1, adjacent 2, and 3:0. Only fertilization with a spermatozoon produced after alternate segregation leads to a genetically normal (46 chromosomes) or balanced (45 chromosomes including one derivative chromosome) karyotype. Fertilization with a spermatozoon produced after adjacent or 3:0 segregation leads to trisomy or monosomy in zygotes, and the majority of these cases are eliminated early, but can also produce offspring with abnormal karyotypes [36, 38, 39]. In prenatal diagnosis, chromosomally imbalanced offspring of RobT carriers appear at a frequency of approximately 7 ± 4% [35, 36]. Viable imbalanced offspring can occur only in carriers of a translocation involving chromosome 13 and/or 21. Moreover, carriers of translocations involving chromosome 14 and/or 15 are at increased risk of the occurrence of uniparental disomy (UPD) in the offspring [38, 40]. UPD involving chromosomes 14 or 15 results in an abnormal phenotype due to the differential expression of paternal and maternal imprinted genes, but the estimated risk of UPD formation is less than 1% [38, 41]. However, foetuses with a normal karyotype with one parent who is a balanced RobT carrier involving chromosome 14 and/or 15 and prenatal cases showing RobT involving these chromosomes should be considered for UPD testing [41].

To date, meiotic segregation patterns have been analysed in sperm from over 200 carriers of different nonhomologous RobTs. On average, the frequency of genetically normal/balanced segregants has been found to be approximately 80% [42, 43]. In contrast, in the carriers of homologous translocations, e.g., der(21;21) or der(13;13), all sperm cells show disomy or nullisomy of a particular chromosome, in agreement with theoretical expectations [44].

It is estimated that among men with different types of reproductive failure, RobTs are detected over 9 times more often than in the general population (i.e., at a frequency of approximately 0.8%) [45, 46]. Among infertile men with normozoospermia, the frequency of detected RobTs is approximately 0.46% [46]. In men with oligozoospermia (including oligoastheno- or/and oligoteratozoospermia), RobTs occur with a frequency of 1.5–3% and are the most common type of the aberration of autosomes (35% of all detected aberrations). In men with azoospermia, RobTs occur at a frequency of 0.2–0.8% [10, 11, 46–48].

The presence of normal sperm concentrations in some RobT carriers raises the question of how these translocations may lead to spermatogenic failure in other carriers. For example, different chromosomal breakpoints, associated with the occurrence of dicentric or monocentric derivative RobT chromosomes, could explain the seminological variability among carriers. However, infertile RobT carriers have been shown to be the sons or brothers of fertile carriers of the same family translocation. It is, therefore, difficult to clearly assess the influence of these translocations on infertility [9, 36]. The observed sperm production impairment may be due to global interactions between the translocated chromosomes and whole-genome instability, rather than the molecular characteristics of the translocation itself [quote from [12]]. Curiously, the frequency of rob(13;14) in the population of normozoospermic and fertile adult men (approximately 0.07%) is very similar to that found in newborns [12].

The reproductive failure of RobT carriers is additionally associated with the fact that in the sperm of many carriers, there is an increase in segregation defects involving chromosomes other than those involved in a particular translocation. To date, the aneuploidy levels of the identified chromosomes have been analysed in sperm from over 100 carriers, and in approximately half of these cases, aneuploidy has been detected [40]. Although data regarding the hyperhaploidy of sperm chromosomes 1, 4, 6, 7, 8, 9, 13, 15, 16, 17, 18, 20, 21, 22, X, and Y have been published, in most of these studies, only a few chromosomes have been examined. The clinical significance of sperm aneuploidy is still the subject of numerous analyses in different groups of patients, and the prevailing view is that even a small increase in sperm aneuploidy may impact fertility [49]. It is estimated that approximately 35% of spontaneous abortions, 4% of stillbirths, and 25% of all zygotes show aneuploidy [50, 51]. On the other hand, the interpretation of the data is hampered by the very high variability of sperm aneuploidy frequencies between carriers, and it is not clear if the type of RobT or its classification into Class 1 or Class 2 makes a difference.

Herein, a retrospective compilation of published data on meiotic segregation patterns and hyperhaploidy in the spermatozoa of rare and common Robertsonian translocation carriers is presented. The data on 213 meiotic segregation patterns and aneuploidy levels in 127 carriers of various heterological RobTs are juxtaposed in this analysis, with a special attention to rare RobTs. The critical analysis of the available data revealed that it is necessary to obtain more results from a considerably larger group of patients, because the data still does not meet the criteria for meta-analysis [52]. As an alternative, the ratio of mean values method (RoM) was used to analyse the data concerning the sperm aneuploidy problem [53]. The RoM analysis was performed on simulated data (i.e., taking into account the average ranges), including normalization against appropriate control values. Considering the division of RobT carriers into the normozoospermic and OAT (oligoasthenozoospermic) subgroups, similar percentages (67% and 70%, respectively) of carriers with elevated levels of aneuploidy were found in the two cohorts. In addition, the analysis of the available data showed that the reason(s) for the differences in semen quality and meiotic segregation disorders between RobT carries are difficult to identify, but this does not indicate a simple connection of a RobT to a Class 1 or Class 2 classification.

Considering the relatively high frequency of occurrence of RobTs and their influence on reproductive failure, further comprehensive chromosomal research on larger groups of RobT carriers will be critical. What remains to be determined is whether more data that are methodologically analogous to what we have collected so far will be sufficient for the sperm chromosomes of RobT carriers to reveal their secrets.

## Methods

### Literature search details

Published papers and abstracts were identified via a thorough computerized literature search of the PubMed electronic database (www.ncbi.nlm.nih.gov). To find articles addressing the meiotic segregation patterns in carriers of RobTs, the following keywords were used: Robertsonian translocation, rare Robertsonian translocation, meiotic segregation, alternate segregant, sperm FISH analysis, and sperm chromosomes. To search for articles addressing sperm aneuploidy in carriers of RobTs, the following keywords were used: Robertsonian translocation, sperm FISH analysis, interchromosomal effect (ICE), and sperm chromosomes aneuploidy.

### Statistical analysis

Data were analysed with RStudio software (version 3.4.4). The normality of the data was checked using the Shapiro–Wilk test. The variability of meiotic segregation patterns and the mean aneuploidy frequencies were compared using the Kruskal–Wallis test adjusted with the Benjamini–Hochberg method and the Friedman test with the Shaffer and Finner corrections. A probability value of *p* ≤ 0.05 was considered to be statistically significant. The heterogeneity of the data concerning the frequencies of aneuploidy was assessed with the R package ‘meta’, function: ‘metagen’. Because the value of the *I*^2^ parameter was > 60, a classic meta-analysis was not possible, so the statistical approach should be interpreted with caution. A lack of sufficient data did not allow us to perform an analysis based on effect size. Therefore, we used the Ratio of Means method (RoM) (which has comparable statistical power to meta-analysis) as an alternative for determining the statistical significance of the differences in sperm aneuploidy frequencies. Using RoM analysis in each case, we automatically achieved normalization with the control group. To include as much published data as possible, in cases where the mean value was not available in a given publication, the average of the range of values was used as an estimation. An RoM value equal to 1 indicates no significant differences from the control values. Values < 1 or > 1 are lower or higher than the control values, respectively; the closer the RoM value is to 1, the smaller is the statistical difference in relation to the control value.

## Results

### Analysis of the data regarding meiotic segregation patterns in carriers of RobTs with special attention to rare cases.

In total, 47 articles published up to January 2020 were identified and considered to have useful information about meiotic segregation patterns in carriers of rare or common RobTs. The analysed data refer to 59 carriers, representing eight types of heterologous rare RobTs with DD, DG, and GG symmetry of the involved chromosomes **(**for individual results, see Supplementary Table S1). All the carriers for whom the analyses were published exhibited reproductive failure. However, the data did not meet the criteria for meta-analysis, since in particular groups of RobTs, the differences in the number of carriers and sperm cells analysed were too large [52]. A summary of the data concerning the mean values of the meiotic segregation results for 59 rare RobT carriers and an additional (for the purpose of comparison) 116 common rob(13;14) and 38 rob(14;21) carriers is presented in Table 1. In the group of rare RobTs, the most published cases (*n* = 17) were available for rob(13;15) and rob(14;22) (*n* = 11), whereas the fewest (*n* ≤ 4) were available for rob(21;22) (*n* = 4), rob(15;22) (*n* = 3), and rob(15;21) (*n* = 1). Substantial differences were also observed for the number of analysed sperm cells; in individual cases, 67 to 10,223 sperm cells were analysed, with an average of 2585 ± 2657. The assessment of the types of segregants in sperm cells is fraught with methodological error, which should be minimized through the analysis of thousands of sperm cells [54]. Unfortunately, only 28% of the results were obtained from a minimum of 3000 sperm cells **(**Table 1 and Supplementary Table S1). It is most likely that oligozoospermia was the reason for the analysis of < 2000 sperm cells in up to 52% of the carriers and < 1000 sperm cells in 22% of the carriers. It is, therefore, probable that at least some of these analyses may include an error in the estimation of the meiotic segregation pattern, making the pattern difficult to assess.

**Table 1:** Summary of published data on the mean values of meiotic segregation results in the spermatozoa of rare RobT carriers^a^ and carriers of common rob(13;14)^b^ and rob(14;21)^c^

rare RobT^a^	Symmetry	No. of carriers	No. of sperm cells: mean value ± SD and range	Type of segregation and [%] of segregants mean value ± SD and range:			
				Alternate	Adjacent	3:0/2n	Other
rob(13;15)	DD	17	**1202 ± **765 67–2978	**76.0** ± 9.6^1^ 50.9–92.8	**22.7** ± 10.1^2^ 6.8–20.4	**0.9** ± 1.1 0.0–4.4	**0.1 ± **0.3 0.0–0.8
rob(14;15)	DD	10	**1962 ± **1432 819–4358	**83.9** ± 8.9^**2**^ 68.3–99.7	**15.5** ± 8.4 0.2–29.8	**0.4** ± 0.7 0.0–1.9	**0.0 ± **0.0 0.0–0.0
rob(21;22)	GG	4	**629** ± 446 149–1016	**79.8** ± 15.5 60.0–97.6	**18.8** ± 12.2 3.4–36.0	**0.5** ± 0.6 0.0–1.2	**1.0 ± **2.0 0.0–4.0
rob(13;21)	DG	7	**6108** ± 4013 1000–10,223	**88.7** ± 3.1^1^ 85.6–94.4	**10.7 ± **2.9^2^ 5.6–14.3	**0.3** ± 0.3 0.0–0.8	**0.1** ± 0.2 0.0–0.3
rob(13;22)	DG	6	**2719** ± 2632 1000–7052	**80.9** ± 6.9 71.9–86.8	**17.1** ± 7.4 9.9–26.5	**0.8** ± 0.6 0.0–1.7	**0.3** ± 0.8 0.0–2.0
rob(14;22)	DG	11	**3174** ± 2342 258–5428	**81.2** ± 7.3 69.4–94.5	**18.1** ± 5.4 5.1–22.0	**0.7** ± 0.5 0.3–1.9	**0.0** ± 0.0 0.0–0.0
rob(15;21)	DG	1	1002	**77.7**	–	–	–
rob(15;22)	DG	3	**2212 ± **1832 118–3517	**86.0 ± **4.5 80.9–89.6	**12.9 ± **4.2 10.0–17.7	**1.2 ± **0.3 1.0–1.4	**0.0** ± 0.0 0.0–0.0
∑ DD		27	**1455** ± 1057 67–4358	**79.2** ± 10.2 50.9–99.7	**19.6** ± 10.2 0.2–49.1	**0.8** ± 1.0 0.0–1.9	**0.1** ± 0.2 0.0–0.8
∑ DG		28	**3681** ± 3114 118–10,223	**83.4 ± **6.7 69.4–94.5	**15.1** ± 5.9 5.1–26.4	**0.7** ± 0.5 0.0–1.7	**0.1** ± 0.4 0.0–2.0
∑ DD,GG,DG		59	**2585** ± 2657 67–10,223	**79.7** ± 9.2 60.0–99.7	**17.7** ± 8.9 0.2–49.1	**0.7** ± 0.8 0.0–1.9	**0.2** ± 0.6 0.0–2.0
Common RobT							
rob(13;14)^b^	DD	116	**1665 ± **1371 78–5985	**79.3** ± 10.2 50.9–99.7	**19.7** ± 10.2 0.2–49.1	**0.8** ± 1.0 0.0–1.9	**0.2** ± 0.2 0.0–0.8
rob(14;21)^c^	DG	38	**1226 ± **986 91–3058	**78.9 ± **11.8 53.3–90.0	**19.1 ± **10.2 7.0–42.9	**1.4 ± **1.1 0.0–3.7	**0.6 ± **0.6 0.0–5.1

Kruskal–Wallis test adjusted with the Benjamin–Hochberg method was used to compare results (*p* ≤ 0.05 was considered to be statistically significant).

Values with significant differences: ^1^: (*p* = 0.029); ^2^: (*p* = 0.032)

^a^References from Suppl. Table S1: [3, 43, 65, 83, 92, 132–149, 150–152, 176]

^b^References: [43, 64–66, 77, 82, 83, 92, 132, 133, 135, 144, 146, 153–162]

^c^References: [42, 43, 65, 66, 82, 132, 133, 138, 139, 144, 146, 148, 162–166]

Taking into account the obtained mean values (Table 1 and Supplementary Table S1), the percentage of normal/balanced spermatozoa (i.e., alternate segregants) in all types of ROBs was higher than 75% overall. However, the mean value for the alternate segregation rate of rob(13;15) (i.e., 76.0%) was significantly lower than the mean values for rob(14;15) (i.e., 83.9%) and rob(13;21) (i.e., 88.7%). Moreover, the mean value for the alternate segregation rate of rob(13;21) was significantly higher than those for rob(13;22) (i.e., 80.9%) and rob(14;22) (i.e., 81.2%) (Table 1). Simultaneously, it should be noted that the extremely small groups of rob(21;22), rob(15;21) and rob(15;22) were probably too small for proper statistical comparison, which may be the reason for the lack of significance regarding their segregation rates in comparison to the other types of ROBs.

The analysis of the individual results of carriers of rare RobTs indicated that the highest percentage of all carriers (46%, *n* = 27) exhibit rates of normal/balanced spermatozoa (alternate segregation) between 75 and 85% (Supplementary Table S1). The percentages of carriers with rates of normal/balanced spermatozoa under 75% and over 85% were 20% (*n* = 12) and 34% (*n* = 20), respectively. Interestingly, all carriers from the rob(13;21) group exhibited alternate segregant rates > 85%, although the possibility cannot be ruled out that this result occurred by chance due to the small size of the group (*n* = 7). It should also be noted that over half (i.e., 58%, *n* = 34) of the carriers of different rare RobTs presented over 80% alternate segregants, but there were only 35% (*n* = 6) with similar results among the carriers of rob(13;15). There were also significant differences in the results between individual carriers, and some individual results were significantly different than the mean values for a given type of RobT (Supplementary Table S2).

Table 1 summarizes the published data concerning the mean values of meiotic segregation in carriers of the common rob(13;14) and common rob(14;21). To date, meiotic segregation patterns have been studied in 116 carriers of rob(13;14) with DD symmetry and 38 carriers of rob(14;21) with DG symmetry. The mean value of rob(13;14) (i.e., 79.3%) for normal/balanced segregation rates was not different from that of rob(14;21) (i.e., 78.9%). Moreover, these mean values for common RobTs were not different from the mean value of the sum of rare RobTs (i.e., 79.7%). However, these common RobT mean values were significantly lower than the mean value for the rare rob(13;21) (i.e. 88.7%) (Table 1).

### Analysis of the data regarding sperm aneuploidy studies in carriers of rare and common RobTs

According to specified criteria, a total of 26 articles published up to January 2020 were identified and considered to contain useful information. The variables analysed were sperm parameters (concentration, motility, and morphology), the frequency of disomy among the selected sperm chromosomes (*n* = 24), and the frequency of diploid (2n) spermatozoa. All RobT carriers for whom estimates were published exhibited reproductive failure (including carriers with normozoospermia). The evaluated data refer to 33 carriers of rare RobTs [including one case of homozygous rob(14;15) and two of rob(21;21)], 77 carriers of common rob(13;14), 17 carriers of common rob(14;21), and 169 control fertile males with a normal karyotype (46, XY). The data for the control groups originated from publications related to aneuploidy in RobT carriers. The total individual results included disomy of chromosomes 1, 4, 6, 7, 8, 9, 13, 15, 16, 17, 18, 20, 21, 22, X, and Y and diploidy (2*n*). The individual results for carriers of rare RobTs, common rob(13;14), rob(14;21), and the control groups are presented in Supplementary Tables S2A-D. The individual results concerned only the hyperhaploidy of chromosomes that were not involved in a given translocation.

### Analysis of differences in mean values

Based on individual data from Supplementary Tables S2A–D, a summary was prepared (Table 2) in which the total aneuploidy (disomy and diploidy) values for all analysed chromosomes with both rare and common types of RobTs and the control groups are juxtaposed. As can be seen in Table 2, the small number of results that we could apply for the analysis of particular types of rare RobTs was of particular importance. Bearing in mind the limitations, based on the data collected in Table 2, the mean aneuploidy levels calculated for the total analysed chromosomes were significantly higher for the group of 33 carriers of rare RobTs (0.51%) as well as for carriers of common rob(13;14) (0.61%) and rob(14;21) (0.42%) than for the control group (0.16%).

**Table 2:** Summary of aneuploidy analysis: mean frequency of hyperhaploidy of analysed sperm chromosomes in the spermatozoa in terms of a carrier of a particular type of rare or common type of Robertsonian translocation (RobT) and in the control group (based of individual data from the Suppl. Tables S2 A–D)

RobT		No. of carriers	No. of results for ∑analysed chromosomes	Mean ± SD (range) [%] per carrier for ∑results for analysed chromosomes
Symmetry	Rare			
DD	**rob(13;15)**	7	45	**0.31**^**1,2,3**^ ± 0.23 (0.05–1.09)
	**rob(14;15)**	4	12	**1.15**^**1,2,4**^ ± 1.76 (0.00–6.26)
GG	**rob(21;22)**	1	2	**0.55**^**1,5**^ ± 0.07 (0.50–0.60)
	**rob(21;21)**	2	10	**0.39**^**1**^ ± 0.20 (0.17–0.87)
DG	**rob(13;21)**	5	14	**0.22**^**2**^ ± 0.20 (0.00–0.57)
	**rob13;22)**	5	20	**0.41**^**1**^ ± 0.54 (0.03–2.50)
	**rob(14;22)**	7	29	**0.66**^**1**^ ± 1.06 (0.00–1.30)
	**rob15;21)**	1	2	**0.65**^**1**^ ± 0.92 (0.00–1.30)
	**rob(15;22)**	1	9	**0.23**^**2**^ ± 0.23 (0.05–0.72)
∑		**33**	**143**	**0.51**^**1,2**^ ± 0.26 (0.00–6.26)
Common				
DD	**rob(13;14)**	77	317	**0.61**^**1,2**^ ± 0.46 (0.00–5.76)
DG	**rob(14;21)**	17	59	**0.42**^**1,2,4**^ ± 0.46 (0.00–1.96)
Control		169	**864**	**0.16**^**1**^ ± 0.13 (0.00–0.85)

Kruskal–Wallis test adjusted with the Benjamin–Hochberg method was used to compare results (*p* ≤ 0.05 was considered to be statistically significant)

^**1**^Mean values are significantly higher than mean control value 0.16 (*p* = 0.00)

^**2**^Mean values are significantly different than mean value 0.61 for common rob(13;14) (*p* = 0.02)

^**3**^Mean value 0.31 for rob(13;15) is lower than mean values 1.15 for rob(14;15) (*p* = 0.002) and 0.66 for rob(14;22) (*p* = 0.035)

^**4**^Mean value 1.15 for rob(14;15) is higher than mean value 0.42 for rob(14;21)(*p* = 0.006)

^**5**^Mean value 0.55 for rob(21;22) is higher than mean value 0.20 for rob(13;21) (*p* = 0.031)

Taking into account the results for individual chromosomes, the threshold of ten results was not reached for any of the individual chromosomes, which seemed to be the minimum number for potentially achieving statistical significance.

Because there were not enough results for particular types of rare RobTs, in subsequent analysis (i.e., Tables 3, 4 and 5), the results for all 33 carriers of rare ROBs were considered as a single group, designated “∑rare RobT”. Unfortunately, only results for chromosomes 1, 7, 8, 9, 18, *X* + *X*, *Y* + *Y*, *X* + *Y,* and diploidy (2*n*) were available for ∑rare RobT, rob(13;14), and rob(14;21) simultaneously.

**Table 3:** Comparison of mean values of hyperhaploidy analysis for particular chromosomes in the spermatozoa of carriers of ∑rare ROBs, common rob(13;14), and common rob(14;21) and control fertile males with normal karyotype (46,XY) (based of the individual data from the Suppl. Tables S2 A-D)

Chromosome	Robertsonian translocation:						Control 46,XY 169 males	
	No. of result for the chromosome No	∑rare* 33 carriers	Common					
			rob(13;14) 77 carriers		rob(14;21) 17 carriers			
		Mean ± SD, (range) %	No	Mean ± SD, (range) %	No	Mean ± SD, (range) %	No	Mean ± SD, (range) %
**1**	4	**0.43** ± 0.21^**5**^ (0.24–0.78)	3	**1.39** ± 0.96^**8**^ (0.33–2.21)	1	**0.67**^**10**^	3	**0.**19 ± 0.05 (0.14–0.25)
**4**	3	**0.28** ± 0.01^**3**^ (0.27–0.28)	0	−	0	–	3	**0.**14 ± 0.05^**1**^ **(**0.09–0.20)
**6**	2	**0.16** ± 0.05 (0.12–0.19)	0	−	0	−	9	**0.13** ± 0.01^**1**^ **(**0.05–0.31)
**7**	4	**0.28** ± 0.22 (0.05–0.54)	10	**0.08** ± 0.09^**8,9**^ (0.01–0.32)	2	**0.10** ± 0.04 (0.07–0.12)	21	**0.11** ± 0.07^**1,2,3**^ **(**0.02–0.16)
**8**	1	**0.08**	11	**0.05** ± 0.04^**5,9**^ (0.00–0.13)	1	**0.07**	10	**0.05** (0.03–0.07)
**9**	9	**0.46** ± 0.42^**6**^^,A^ (0.17–1.50)	3	**0.16** ± 0.09 (0.08–0.26)	1	**0.16**	14	**0.15** ± 0.04^**1**^^,A^ **(**0.04–0.25)
**13**	6	**0.70** ± 0.63^**4**^^,B^ (0.00–1.39)	0	−	9	**1.00** ± 0.65^**11**^^,B^ (0.12–1.96)	79	**0.14 ± **0.09^**1**^^,^^**3**^^,B^ **(**0.03–0.47)
**15**	3	**0.24** ± 0.08 (0.14–0.29)	2	**1.37** ± 0.71^**8**^^,C^ (0.87–1.87)	0	−	10	**0.26** ± 0.22^**1**^^,C^ **(**0.09–0.51)
**16**	4	**0.49** ± 0.30 (0.18–0.87)	0	−	0	−	4	**0.25**^**2**^ ± 0.08 **(**0.09–0.30)
**17**	1	**0.27**	0	−	0	−	1	**0.20**
**18**	24	**0.28** ± 0.43^D^ (0.00–1.29)	72	**0.36** ± 0.62^**8**^^,^^**9**^^,D^ (0.00–3.20)	16	**0.29** ± 0.20^**11**^^,D^ (0.00–0.86)	142	**0.11** ± 0.11^**1,2,3,4**^^,D^ **(**0.00–0.55)
**20**	3	**0.79**^**3**^± 0.78 (0.27–1.68)	0	−	0	−	3	**0.15** ± 0.04 **(**0.10–0.19)
**21**	14	**0.40** ± 0.31^**7**^^,E^ (0.00–1.09)	40	**0.71** ± 0.86^**9**^^,E^ (0.00–4.72)	0	−	120	**0.21** ± 0.14^**3**^^,E^ **(**0.04–0.80)
**22**	2	**0.27** ± 0.04 (0.24–0.29)	12	**0.56** ± 1.02 (0.00–2.96)	0	−	26	**0.20** ± 0.25^**4**^ **(**0.00–0.85)
**XX**	12	**0.18** ± 0.15^**3,4,5,6,7**^^,F^ (0.00–0.40)	29	**1.04** ± 1.68^F^ (0.00–5.76)	4	**0.06** ± 0.02^**10,11**^ (0.00–0.18)	76	**0.11** ± 0.10^1,2,3,4,F^ **(**0.00–0.71)
**YY**	12	**0.23** ± 0.18^**3,4**^^,G^ (0.05–0.59)	29	**0.57** ± 0.74^**G**^ (0.00–2.50)	4	**0.06** ± 0.00^**10,11**^ (0.00–0.09)	81	**0.12** ± 0.11^**1,2,3,4**^^,G^ **(**0.00–0.71)
**XY**	12	**0.25** ± 0.14^**3,4**^ (0.10–0.53)	29	**0.30** ± 0.31^**8**^ (0.00–1.16)	4	**0.29** ± 0.17 (0.16–0.52)	105	**0.21** ± 0.19^**3**^ **(**0.00–0.65)
**2n**	27	**1.04** ± 1.66^H^ (0.00–6.26)	77	**0.68** ± 1.08^H^ (0.00–6.46)	17	**0.50**^**11**^ ± 0.48^H^ (0.00–1.42)	157	**0.16** ± 0.19^3,H^ (0.00–0.50)

*∑rare RobT = rob(13;15), rob(14;15), rob(21;22), rob(21;21)^@^, rob(13;21), rob(13;22) rob(14;22), rob(15;21), rob(15;22). ^@^Homologous RobT. Kruskal–Wallis test adjusted with the Benjamin-Hochberg method was used to compare results;* p* ≤ 0.05 was considered to be statistically significant

Statistical differences in mean values of hyperhaploidy levels of individual chromosomes between different ROBs: ∑rare, rob(13;14), common (14;21) and control group:

^A^For chromosome 9 control mean value 0.15 is differ than mean value 0.46 for ∑rare (* p* = 0.011);

^B^For chromosome 13 control mean value 0.14 is differ than mean values 0.70 for ∑rare and 1.00 for rob(14;21) (* p* = 0.00);

^C^For chromosome 15 control mean value 0.26 is differ than mean value 1.37 for rob(13;14) (* p* = 0.001);

^D^For chromosome 18 control mean value 0.11 is differ than mean values 0.29 for rob(14;21), 0.36 for rob(13;14)and 0.28 for ∑rare (* p* = 0.00);

^E^For chromosome 21 control mean value 0.21 is differ than mean values 0.71 for rob(14;21) and 0.40 for ∑rare (* p* = 0.00);

^F^For XX control mean value 0.11 is differ than mean values 1.04 for rob(13;14) (p = 0.000) and 0.18 ∑rare (p* p*= 0.039);

^G^For YY control mean value 0.12 is differ than mean values 0.57 for rob(13;14) and 0.23 for ∑rare (* p* = 0.00);

^H^For 2n control mean value 0.16 is differ than mean values 0.50 for rob(14;21); 0.68 for rob(13;14) and for ∑rare 1.04 (* p* = 0.00)

Statistical differences between mean values of hyperhaploidy levels for individual chromosomes:

Control: ^**1**^mean values lower than mean value 0.26 for chromosome 15 (* p* = 0.02); ^**2**^mean values lower than mean value 0.25 for chromosome 16 (p = 0.02); ^**3**^mean values lower than mean value 0.21 for chromosomes 21 and XY (= 0.02); ^**4**^mean values lower than mean value 0.20 for chromosome 22 (*p* = 0.03)

∑Rare: ^**3**^mean values lower than mean value 0.79 for chromosome 20 (*p* = 0.00); ^**4**^mean values lower than mean value 0.70 for chromosome 13 (*p* = 0.03); ^**5**^mean values lower than mean value 0.43 for chromosome 1 (*p* = 0.00); ^**6**^ mean values lower than mean value 0.46 for chromosome 9 (p = 0.05); ^**7**^ mean values lower than mean value 0.40 for chromosome 21 (*p* = 0.04)

rob(13;14): ^**8**^mean values lower than mean values 1.39 for chromosome 1 and also 1.37 for chromosome 15 (p = 0.03); ^**9**^mean values lower than mean value 0.71 for chromosome 21 (*p* = 0.03);

rob(14;21): ^**10**^ mean values lower than mean value 0.67 for chromosome 1 (*p* = 0.00);^**11**^ mean values lower than mean value 1.0 for chromosome 13 (*p* = 0.04)

**Table 4:** Summary of RoM analysis of data on chromosome aneuploidy frequencies in the spermatozoa from RobT carriers performed on the base of individual data from Supplementary Tables S2 A–D. The RoM values equal to 1 indicates no significant differences from the control values. Values < 1 or > 1 are lower or higher than the control values, respectively; the closer the RoM value is to 1, the smaller is the statistical difference in relation to the control value

Chromosome	∑rare RobT 33 carriers				rob(13;14) 77 carriers				rob(14;21) 17 carriers			
	RoM values				RoM values				RoM values			
	Min	Max	Mean	Median	Min	Max	Mean	Median	Min	Max	Mean	Median
**1**	1.3	1.7	1.5	**1.5**	–	–	–	–	–	–	–	–
**4**	1.9	1.9	1.9	**1.9**	–	–	–	–	–	–	–	–
**6**	0.9	0.9	0.9	**0.9**	–	–	–	–	–	–	**–**	–
**7**	1.7	1.7	1.7	**1.7**	0.3	2.7	1.1	**0.8**	0.8	2.3	**1.5**	**1.5**
**8**	1.6	1.6	1.6	**1.6**	0	2.6	1.0	**0.8**	− 1.4	− 1.4	**− 1.4**	**− 1.4**
**9**	1.2	2.2	1.7	**1.7**	0.6	0.6	0.6	**0.6**	1.2	1.2	**1.2**	**1.2**
**13**	1.1	13.6	6.0	**5.2**	**–**	–	–	–	0.9	7.8	**4.7**	**4.3**
**15**	1.6	1.6	1.6	**1.6**	1.7	3.7	2.7	**2.7**	–	–	**–**	–
**16**	1.0	2.0	1.5	**1.7**	_	–	–	–	–	–	**–**	–
**17**	1.4	1.4	1.4	**1.4**	_	_	–	–	–	–	**–**	–
**18**	0	10.8	2.8	**2.7**	0	26.7	3.7	**1.7**	0	7.2	**3.3**	**3.0**
**20**	1.8	2.7	2.3	**2.3**	–	–	–	–	–	–	**–**	–
**21**	0	14.1	3.0	**1.9**	0.2	8.9	2.3	**1.5**	–	–	**–**	–
**22**	–	–	–	–	0	5.1	1.9	**1.6**	–	–	**–**	–
**XX**	0.1	7.0	2.4	**1.9**	0.3	29.5	6.8	**2.1**	0.7	2.0	**1.5**	**1.8**
**YY**	0.1	14.8	4.6	**2.4**	0	13.3	4.4	**3.6**	1.1	2.3	**1.7**	**1.6**
**XY**	0.1	7.0	2.5	**1.9**	0	12.9	2.0	**1.2**	2	3.6	**2.9**	**3.1**
**2n**	0	69.6	11.4	**2.8**	0	215.3	8.3	**1.9**	0	35	**6.4**	**4.8**
Average of ROM values	0.9 ± 0.7	18.0 ± 21.4	2.9 ± 2.5	**2.1** ± 0.9	0.3 ± 0.5	29.2 ± 59.6	3.2 ± 2.4	**1.7** ± 0.9	0.9 ± 0.6	6.6 ± 10.9	**2.3 ± 2.1**	**2.2** ± 1.4

**Table 5:**
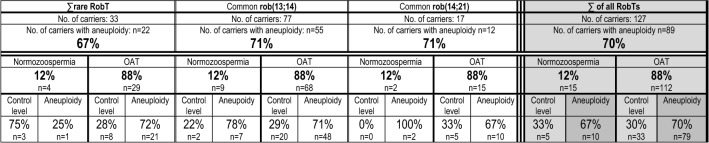
The percentage of Robertsonian translocation (RobT) carriers with elevated aneuploidy level of analyzed sperm chromosomes taking into account ejaculate parameters: normozoospermia in contrast to OAT (oligoasthenozoospermia with different intensity) (based of individual data from the Suppl. Tables S2 A-D)

As seen from the results for particular chromosomes presented in Table 3, in the ∑rare RobT group, the mean frequency of hyperhaploidy ranged from 0.16% (for chromosome 6) to 1.04% for 2n (diploidy). However, the criterion of ten results was met for the mean values for chromosomes 18, 21, *X* + *X*, *Y* + *Y*, *X* + *Y,* and 2*n* (diploidy). For chromosomes 18 (0.28%), 21 (0.40%), *X* + *X* (0.18%), *Y* + *Y* (0.23%), and 2n (1.04%), the hyperhaploidy level was markedly higher than the control mean values (0.11%, 0.21%, 0.11%, 0.12%, and 0.16%, respectively).

In the rob(13;14) group, the mean frequency of hyperhaploidy ranged from 0.05% (for chromosome 8) to 1.37% and 1.39% (for chromosomes 15 and 1, respectively). The criterion of ten results was met for the mean values for chromosomes 7, 8, 18, 21, 22, *X* + *X*, *Y* + *Y*, *X* + *Y,* and 2*n* (diploidy). For chromosomes 7 (0.08%), 8 (0.05%), 18 (0.36%), 21 (0.71%), 22 (0.56%), *X* + *X* (1.04%), *Y* + *Y* (0.57%), and 2*n* (0.68%), the hyperhaploidy level was higher than the control mean values (0.11%, 0.05%, 0.11%, 0.21%, 0.20%, 0.11%, 0.12%, and 0.16%, respectively).

In the rob(14;21) group, the mean frequency of hyperhaploidy ranged from 0.6% (for chromosomes X and Y) to 1.0% (for chromosome 13). The criterion of ten results was met for the mean values for chromosome 18 and 2*n* (diploidy). For chromosome 18 (0.29%) and 2n (0.50%), the hyperhaploidy level was higher than the control mean values (0.11% and 0.16%, respectively).

In the control group, the mean frequency of hyperhaploidy ranged from 0.05% (for chromosome 8) to 0.26% (for chromosome 15). In the control group, ten results were only obtained for chromosomes 7, 8, 9, 13, 15, 18, 21, 22, *X* + *X*, *Y* + *Y*, *X* + *Y,* and 2*n* (diploidy). Among these chromosomes, the mean hyperhaploidy levels of chromosomes 15 (0.26%), 21 (0.21%), 22 (0.20%), and *X* + *Y* (0.21%) were higher than those of the other chromosomes.

The analysis of the control group regarding the frequency of aneuploidy for particular chromosomes (Supplementary Table S2D) revealed that the control results originating from different laboratories differed significantly [49]. The reasons for such differences are difficult to determine, although they undoubtedly may be influenced by factors such as significant differences in both the number of analysed cells and the size of the studied group [49, 54–58]. The differences related to statistical approaches are also of great importance [59]. As a consequence, the results for individual patients regarding the frequency of aneuploidy obtained in a particular laboratory must be compared with baseline aneuploidy values (i.e., aneuploidy thresholds) established for the corresponding control group [59]. For this reason, in Supplementary Tables S2A–C, the individual results for the examined chromosomes indicated with a bold asterisk represent significantly different values versus the control data from a particular report and do not refer to mean values calculated from all cited publications. These differences were the most evident, at 49.0% (143/70*), for the group of rare ROBs, whereas the corresponding values for the carriers of rob(13;14) and rob(14;21) were 40.4% (317/128*) and 45.8% (59/27*), respectively, but these differences were not statistically significant (*Z* test, *p* < 0.05**).**

### Analysis of the differences in RoM (Ratio of Means method) values

All the listed limitations concerning the comparison of mean chromosome aneuploidy values were the reasons that an alternative statistical analysis had to be applied. These data did not meet the criteria for meta-analysis [52], but it was possible to use RoM approach instead. The RoM method exhibits similar statistical power to meta-analysis [53]. The calculation of RoM values is based on simulated data with normalization to appropriate control values. Table 4 shows the results of the RoM analysis of the differences in sperm aneuploidy frequencies performed on the basis of data from Supplementary Tables S2A-D. An RoM value equal to 1 indicates no significant differences from the values for the control group. Values < 1 or > 1 are lower or higher, respectively, than the control values; the closer the RoM value is to 1, the smaller the statistical difference is in relation to the control. The data in Table4 show that for the majority of the examined chromosomes, the RoM min and max values differ enormously, which means that the median RoM values could be more appropriate for the interpretation of the results than the RoM means. In the case of carriers of the ∑rare RobT group, the RoM median values were > 1 for almost all analysed chromosomes, with the exception of chromosome 6. Values > 2.0 (i.e., 100% higher than the control value) were found for chromosomes 13, 18, 20, *Y* + *Y,* and 2*n* (diploidy); the highest value of 5.2 was found for chromosome 13. In the case of carriers of rob(13;14), RoM median values > 1 were not found for chromosomes 7, 8, and 9; values > 2.0 were found for chromosomes 15, *X* + *X*, and *Y* + *Y* with the highest value of 3.6 being found for chromosome *Y* + *Y*. For the carriers of rob(14;21), RoM median values > 1 were found for almost all analysed chromosomes, with the exception of chromosome 8. Values > 2.0 were found for chromosomes 13, *X* + *Y* and 2*n* (diploidy), with the highest value of 4.8 being found for 2*n* (diploidy).

Unfortunately, disomy results for only chromosomes 7, 8, 9, and 18, the sex chromosomes, and 2*n* (diploidy) were simultaneously available for ∑rare RobT, common rob(13;14), and common rob(14;21). Among these chromosomes, the highest median RoM values were associated with the sex chromosomes and 2*n* (diploidy), with the highest RoM value of 4.8 being calculated for the 2n results for rob(14;21) carriers, while a value of 3.6 was calculated for the *Y* + *Y* results for rob(13;14) carriers. For both ∑rare RobT and rob (14;21) carriers, the highest RoM values were calculated for chromosome 13 (5.2 and 4.3, respectively) and 2n (2.8 and 4.8, respectively). In turn, for rob(13;14) carriers, the highest RoM values were calculated for chromosome 15 (2.7) and *Y* + *Y* (3.6).

Interestingly, the average values calculated from the median RoM values for all analysed chromosomes were very similar for ∑rare RobT and common rob(14;21) carriers (i.e., 2.1 ± 0.9 and 2.2 ± 1.4, respectively), but they were approximately 20% higher than average value of 1.7 ± 0.9 for rob(13;14) carriers (Table 4).

Table 5 presents a summary of the aneuploidy analyses considering ejaculate parameters (normozoospermia versus a pathological spermiogram). Because oligoasthenoteratozoospermia (OAT) is the most frequent disorder among RobT carriers, in Table 5, the group of carriers with abnormal spermiograms was referred to as the OAT group, regardless of the number or the type of anomalies involved. In all three groups: ∑rare RobT, common rob(13;14), and rob(14;21), the proportion of all carriers exhibiting a significantly higher frequency of sperm aneuploidy for at least one of the analysed chromosomes was similar, at 67%, 71%, and 71%, respectively (including both carriers with normozoospermia and OAT) (Table 5). At the same time, the data showed that among all RobT carriers included in Supplementary Tables S3A–C, only 12% were normozoospermic, and 88% exhibited abnormal spermiograms. Among this 88% of carriers with OAT, an average of 70% exhibited detectable aneuploidy [similar results of 72%, 71%, and 67% were obtained for the ∑rare RobT, common rob(13;14) and rob(14;21) carriers, respectively]. It seems that the numbers of RobT carriers with normozoospermia in the groups with particular types of RobTs are too small for similar estimations to be performed (despite being presented in Table 5). However, considering the total RobT carriers with normozoospermia, it can be stated that aneuploidy was found in 67% of cases, which was a similar result to that for carriers with OAT (70%) (Table 5) (sic!).

## Discussion

Based on the current literature, it is estimated that fertile individuals include approximately 0.07–0.08% of RobT carriers, while among men exhibiting reproductive failure, the percentage of ROB carriers is approximately 0.2–0.8% [10–12, 45–48]. However, a few studies have determined the percentage of infertile men among RobT carriers. It should be mentioned that on the basis of data from 101 pedigrees of common rob(13;14) carriers identified for a wide range of reasons (the birth of a child with congenital anomalies, recurrent pregnancy loss, or fertility problems), it was estimated that the infertility risk of male carriers in this sample was only 11.3%, but this could be underestimated due to the use of a pedigree ascertainment correction [35]. However, the percentage of infertile men among the carriers of both rare and common RobTs can also be estimated indirectly on the basis of general population data. It is estimated that among 5% of infertile males in the reproductive age, about 0.8% (i.e., ± 520.000 individuals) are infertile RobT carriers [60]. On the other hand, the frequency of RobTs in the population of fertile men (approximately 0.07%) [12] indicates that there are approximately 865.000fertile Rob carriers, accounting for ± 50% of the total male RobT carriers of reproductive age. Therefore, when analysing data on the sperm chromosomes of RobT carriers, one has to bear in mind that these data mainly come from RobT carriers who sought medical diagnosis and assistance in achieving pregnancy, whereas we have practically no data for the remaining ± 50% of RobT carriers. Perhaps, the lack of analogous research on fertile carriers is “the missing link” that makes it difficult or even controversial to interpret the large variations reported in different studied populations of infertile individuals.

In the present study, we juxtaposed and summarized the available data from the literature regarding the sperm chromosomes (meiotic segregation patterns and aneuploidy levels) of carriers of various heterological RobTs, with special attention paid to rare RobTs.

### Data regarding meiotic segregation patterns

According to the data presented in Table 1**,** meiotic segregation patterns have been identified in 59 carriers of rare and 154 carriers of common nonhomologous RobTs (∑ = 213).

In the reports published to date, the authors have indicated that the frequency of genetically normal/balanced segregants is approximately 80% on average in carriers of different nonhomologous RobTs [42]. This is confirmed in the data presented in Table 1: the mean values calculated for the rates of normal/balanced sperm cells for ∑rare RobT and both common rob(13;14) and rob(14;21) carriers did not differ and reached almost 80% (*p* < 0.05, Kruskal–Wallis test adjusted with the Benjamini–Hochberg correction). The mean rates of normal/balanced sperm cells associated with all types of rare RobTs were higher than 75%. The group of rob(13;15) carriers showed the lowest mean rate of normal/balanced sperm cells (76.0%) (significantly lower than mean rates for rob(13;21) and rob(14;15)). The highest mean rate of normal/balanced sperm cells (88.7%) was found for rob(13;21) carriers [significantly higher than mean rates for rob(13;22), rob(14;22), ∑rare RobT, common rob(13;14), and common rob(14;21)] (Table 1).

Somewhat different statistical results from the published data concerning segregation pattern analysis were presented by Lamotte et al*.* [43]. It seems that the indicated differences reflected the consequences of different statistical tests: Lamotte et al. [43] used a *t* test (*p* ≤ 0.05 was considered significant) for the comparison of pairs of groups, while we used the Kruskal–Wallis test adjusted with the Benjamini–Hochberg method for the comparison of all groups.

It has been suggested that the prevailing number of normal/genetically balanced sperm cells results from the fact that trivalent pairing of chromosomes occurs in the pachytene stage in the *cis* configuration, which facilitates alternative segregation [61–63]. Luciani et al. [62] performed meiotic cytogenetic analysis on testicular biopsies and indicated that trivalents always occurred in *cis* configuration. According to many authors, the analysed data can indicate “homogenous segregation behaviour” of RobTs independent of the chromosome pairs involved [42, 64–66]. While we concur with this general opinion in principle, it must be noted that some meiotic segregation patterns, both among the individual results (Supplementary Table S2) and between the average records for different translocations (Table 1), showed statistically significant differences (*p* < 0.05, Kruskal–Wallis test). Considering the individual results (Supplementary Table S2), it should be noted that the frequency of alternate segregants of close to 90% found in approximately 20% of RobT carriers appears to be even higher than the theoretically expected frequency following the favoured *cis* configuration in the pachytene stage [62]. When interpreting these results, it should be noted that ejaculated sperm cells are the final product of spermatogenesis and, therefore, provide information only about those meiotic segregants that have not been eliminated at earlier stages of spermatogenesis. Therefore, it should be taken into account that the “overrepresentation” of alternate segregants may partly be a consequence of the elimination of many of unbalanced segregants at meiotic checkpoints and/or during spermiogenesis [67–70]. In turn, to explain whether the rare cases (only a small percentage according to data from Supplementary Table S2) exhibiting a percentage of alternate segregants below 66% are the consequence of a *trans* configuration in the pachytene stage, it would be necessary to perform an analysis of trivalents in such individuals. Without a doubt, what would greatly help in understanding of the results concerning the reduction of spermatocytes and/or the number of unbalanced gametes better, would be a proper assessment of proportion of the cis/trans configuration during the meiotic prophase I. Such an assessment is not possible in most studies on human RobT cases.

## Limitations of three-colour FISH analysis

At the same time, the possibility cannot be excluded that some of the differences in the individual results **(**Supplementary Table S2) may represent an overestimation of the frequency of some FISH signals found in sperm, which could be the result of several problems associated with the FISH method itself. Segregation pattern analysis by three-colour FISH with simultaneous application of centromeric and telomeric probes (which is the most commonly applied) has several limitations. Sperm cells developing at 3:0 segregation with no FISH signals cannot be discriminated from sperm artefacts. The rate of these segregants might be estimated by doubling the number of spermatozoa with FISH signals derived from all three chromosomes involved in a particular RobT. However, there is still inherent error in such estimates, because similar FISH signals may arise from diploid (2*n*) spermatozoa. Moreover, three-colour FISH requires the simultaneous application of centromeric and subtelomeric probes of different sizes as well as a good hybridisation efficiency and signal intensity. In some sperm nuclei, intensive signals from centromeric probes may mask signals from telomeric probes. Furthermore, the lack of some signals interpreted as an absence of a defined chromosome in sperm nuclei cannot be distinguished from a hybridisation artefact. Awareness of these limitations allows the determination of an estimation error of at least a few percent [71, 72]. These limitations can be minimized when analysing a large number of sperm cells (e.g., > 3000). Thus, the fact that such a criterion is met by only approximately 30% of the published data (see Supplementary Table S2) significantly hinders the interpretation of the available data. Additional consternations may arise from the observation made by Anton et al*.* [72] that meiotic segregation patterns are more homogeneous among series of individuals analysed within a single laboratory, even when different kinds of Robertsonian translocations are compared. This would indicate that a certain amount of the variability observed within RobTs could be more closely related to technical aspects (e.g., the particular scoring criteria established in the studied groups) than to the specific cytogenetic characteristics of the rearrangements [72].

Moreover, under the FISH method with simultaneous application of centromeric and telomeric probes, spermatozoa with normal or balanced chromosomes (both results of alternate segregation) exhibit the same fluorescent phenotype and cannot be distinguished. This limitation is minimized, assuming that normal and balanced segregants develop in a 1:1 proportion. Although there are reports of a prevailing number of genetically balanced embryos [36], for most carriers of nonhomologous RobTs, the numbers of offspring with normal and balanced karyotypes are similar, which is consistent with theoretical expectations [73].

Considering the data as a whole (Table 1), it can be stated that the differences (and similarities) in the meiotic segregation patterns between the different types of RobTs exhibit no relationship to either the type of DD/GG/DG symmetry of the involved chromosomes or the Class 1 or Class 2 classification based on molecular research (details in the “Introduction”).

### Data analysis of spermiograms in RobTs carriers

Since the first report by Plymate et al. [74], many studies have shown that most infertile RobT carriers exhibit altered spermatogenesis, i.e., they exhibit oligoasthenoteratozoospermia with varying degrees of intensity [43, 75–77]. Additionally, unusual ultrastructural sperm anomalies related to sperm immaturity are observed [78]. It is theoretically assumed that spermatogenic impairment can be a consequence of a chromosomal imbalance generated as a result of a trivalent configuration during meiotic prophase I [6]. The accurate estimation of the percentage of infertile RobT carriers with normal spermiograms presents some difficulties, because the published calculations differ greatly, which may suggest the random sampling of too few analysed patients, e.g., 14.3% among 63 analysed carriers [79], 25.7% among 35 carriers [80], or 30.8% among 13 carriers [81]. In earlier reports, it was estimated that among RobT carriers for whom meiotic segregation patterns and ejaculate/spermiogram analysis data were available at the same time (i.e., approximately 80% of published data); approximately 18% of the patients were normozoospermic [43]. Some authors have indicated that RobT carriers with normal sperm parameters and those with OAT display similar frequencies of genetic normal/balanced sperm cells (80% on average), suggesting that the impairments of spermatogenesis and meiotic segregation patterns are most likely independent features [65, 66]. In contrast, Lamotte et al*.* [43] intriguingly reported that normozoospermic RobT carriers display significantly higher rates of normal/balanced sperm cells (85%) than carriers with seminogram anomalies (81.3%), regardless of the number or type of anomalies involved (*p* < 0.01 by *t* test). At the same time, the small numbers of patients exhibiting particular types of rare RobTs are inadequate for reliably estimating whether the percentage of normozoospermic patients is similar between different types of RobTs [43].

### Frequency of aneuploidy

Beginning with some of the earliest research on sperm chromosomes using the FISH method, attention was drawn to the fact that more than half of the carriers of different RobTs exhibited aneuploidy of some sperm chromosomes unrelated to those involved in a particular translocation [6, 82, 83]. Most often, a limited number of chromosomes have been selected for these analyses, particularly due to the high frequency of trisomy occurring in living offspring [84]. The high frequency of aneuploidy in sperm cells of RobT carriers is particularly intriguing, because it may indicate the occurrence of the so-called “interchromosomal effect” (ICE). The term ICE is used to describe a phenomenon in which the presence of chromosomes involved in a translocation affects the aneuploidy of the chromosomes that are not involved in the translocation [85]. This phenomenon was first postulated by Lejeune [5] and Aurias et al. [86]. A possible cause of ICE was indicated by Luciani et al. [62], who found an association of the appearance of trivalents and sex vesicles in most of the nuclei at the pachytene stage in an infertile rob(13;14) carrier. It was suggested that this association could produce severe spermatogenetic impairment and be related to heterosynapsis, which might appear in asynaptic sites typical of trivalent formation in prophase I. Because asynaptic regions undergo heterochromatinization and gene silencing, heterosynapses can potentially serve as a rescue mechanism for the effects of asynapsis [67, 87]. However, it may simultaneously lead to aberrant recombination, which is a predisposing factor for non-disjunction at anaphase I, because the number and distribution of chiasmata are crucial for bivalent orientation at the meiotic spindle [84, 88–91]. Another consequence of the presence of heterosynaptic regions at prophase I could be altered chromosome positioning, which may be maintained at least until the metaphase I stage. A chromosome territoriality alteration in the presence of RobTs has in fact been described in metaphase I in human spermatocytes [92]. This alteration led to changes in mature spermatozoa, and altered positioning of NORs (nucleolar organizing regions) was found, which indicated that in RobT carriers (both rare and common), there can be perturbations in the nuclear organization of sperm acrocentrics [93]. These alterations in the internuclear sperm organization can be attributed to changes in the expression profile of paternal alleles in the embryo, which may suggest that the disturbed topology of sperm chromosomes could be an additional factor in reproductive failure. The key to understanding this issue would undoubtedly be to clarify whether chromosome positional variations in infertile carriers are the cause or the consequence of impaired spermatogenesis [92, 94–97].

The interesting observation that aneuploidy occurs preferentially in imbalanced (adjacent) segregation products has been made [83]. The relatively low frequency of aneuploidy in normal/balanced alternate segregants indirectly confirms the speculation that meiotic checkpoints may detect meiotic abnormalities. However, in some situations, resulting from excess anomalies, for example, the elimination of the imbalanced cells by checkpoint-controlled meiotic arrest is not very efficient [67, 69, 98].

Although it has been suggested that heterosynapsis can occur preferentially between trivalents and sex chromosomes as well as acrocentric chromosomes, it is not clear which of the sperm chromosomes are associated with the phenomenon of aneuploidy most frequently [47, 62, 63, 90, 99–102]. It is also unclear which of the types of RobTs are particularly vulnerable to the ICE effect. First, it has been suggested that a high frequency of aneuploidy can be restricted to translocation carriers with abnormal spermiograms [103, 104]. As shown in Tables 1 and 3, aneuploidy frequencies have been tested in 33 carriers of rare and 94 carriers of common RobTs (127 cases in total). In all three groups of RobT carriers (Table 5) [∑rare, common rob(13;14), and common rob(14;21)], the percentages of carriers with a high level of aneuploidy of at least one of the tested chromosomes were similar, at approximately 70% (without taking into account spermiograms), which seems to be higher than previous estimates (greater than 50%) [6, 82, 83]. Interestingly, in all three groups of RobT carriers (Supplementary Tables S2A–C), 12% of carriers were normozoospermic, while 88% exhibited abnormal spermiograms. Generally, among all RobT carriers with abnormal seminograms and normozoospermic carriers, the percentages of patients with detectable aneuploidy were similar (70% and 67%, respectively) (Table 5). Significantly, these individual data showed (Supplementary Tables S2A–C) that among the 127 RobT carriers examined for the aneuploidy frequency, only 4% of carriers (i.e., 5/127) were simultaneously normozoospermic and exhibited normal aneuploidy levels. However, it should be emphasized once again that these data concern only RobT carriers with reproductive failure and that we do not have adequate data from the sperm chromosomes of fertile normozoospermic carriers.

The juxtaposition and RoM analysis of the available data from a relatively large group of 127 patients related to particular chromosomes showed that only the results for disomy of chromosomes 7, 8, 9, 18, the sex chromosomes, and diploidy (2n) were simultaneously available for all three groups of ∑rare RobT, common rob(13;14) and common rob(14;21) (Table 4). The RoM values for the ∑rare RobT and common rob(14;21) groups seem to exhibit more similarities compared to the common rob(13;14) group. Interestingly, the results for the rob(14;21) group were characterised by a particularly high RoM value for diploid (2*n*) and disomic XY sperm cells, which was approximately a 50% higher than the value for the rob(13;14) group. In contrast, the rob(13;14) group was characterised by 25% and 40% less visibility of XX or YY sperm cells compared to the ∑rare RobT and common rob(14;21) groups, respectively. It is important to note that the disomy of sperm autosomes and diploidy (2n) may be consequence of mis-segregation in both the first and second meiotic divisions. In contrast, XY sperm cells originate as a result of non-disjunction events at the first meiotic division, whereas XX or YY sperm cells are derived from meiosis II errors, involving the mis-segregation of sister chromatids. Such a significant contribution of defects during the second meiotic division also indicates serious disorders in meiotic progression associated with general spermatogenetic defects and cannot be attributed only to the interchromosomal phenomenon [105].

The data collected for acrocentric chromosomes 13, 15, and 21 are potentially very interesting due to the tendency of these chromosomes to form heterosynapses, but were available simultaneously only for two of the three groups of RobT carriers (Table 4). Thus, these data leave a large margin of error, especially in the context of the question of which of the chromosomes are associated with the phenomenon of aneuploidy particularly often. The results of the RoM analysis (Table 4) indicate the highest level of aneuploidy of chromosome 13, which was higher than that of chromosomes 15, 21, and 22 (no data available for chromosome 14). The results for the common rob(13;14) group highlighted a high level of disomy of chromosome 15 (2.7 × higher than the control level), coexisting with a high level of disomy YY (3.6 × higher than the control level). This finding is interesting in the context of observations, indicating that bivalent 15 regularly associates with sex vesicles in normal male meiosis, probably due to its high-sequence homology with Yq heterochromatin [106]. However, for the ∑rare RobT group, an analogous association was not found (Table 4).

### The ICE problem

Do the data on aneuploidy summarized in Table 5 mean that approximately 70% of infertile RobT carriers truly exhibit ICE? One can get the impression that for many researchers, each case of elevated hyperhaploidy detected in the sperm cells of RobT carriers is synonymous with true ICE; however, it is doubtful whether this is in fact the case. As seen from the individual data (Supplementary Tables S2A–B), people who carry this type of RobT (and exhibit abnormal spermiograms) often show increased hyperhaploidy for different chromosomes; therefore, it is difficult to argue that an ICE effect appears in relation to each case. Moreover, infertile men with a normal somatic karyotype but abnormal spermiograms exhibit two- to three-fold increase in the rate of chromosomal aneuploidy compared to fertile controls, and great interindividual variation exists [49, 107, 108]. Therefore, for some RobT carriers with abnormal spermiograms, the possibility cannot be excluded that a high frequency of aneuploid chromosomes may originate from spermatogenetic disorders and meiotic disturbance, regardless of the occurrence of translocation itself. Even in seemingly obvious cases of normozoospermic RobT carriers, it is difficult to say that each case of observed aneuploidy is a consequence of ICE. Approximately 10% of fertile healthy men with normal somatic karyotypes also show significantly higher rates of aneuploidy of sperm chromosomes than the rest of the population. These cases may indicate that aneuploidy does not always indicate reproductive failure [56, 109, 110]. Nevertheless, in summarizing the observations about the incidence of hyperhaploidy in RobT carriers, it can be noted that the data do not exclude the possibility that aneuploidy may be both a consequence and an additional cause of impaired spermatogenesis. More observations on the synaptonemal complexes of RobT carriers could clearly help to address these dilemmas; the most commonly used approach of sperm cell FISH will not answer all the remaining questions, since we can observe only the effects of the disorder and not its mechanism in ejaculated sperm cells.

The three analysed groups of carriers with reproductive failure [carriers of rare RobTs, common rob(13;14), and common rob(14;21)] did not differ in terms of the percentages of carriers with abnormal spermiograms (approximately 88%) or elevated levels of sperm aneuploidy (approximately 70%) or the mean rate of normal/balanced sperm cells (close to 80%). Therefore, it seems that semen quality and meiotic chromosome segregation are not exclusively dependent upon the type of RobT (Class 1 or Class 2).

### Other factors: heteromorphisms and sperm architecture

Nevertheless, it should be considered that additional defects/factors may also contribute to decreased fertility in RobT carriers. Such factors could include the presence of morphological variants (heteromorphisms) of acrocentric chromosomes. It is estimated that heteromorphisms in the general population occur with a frequency of 2–5% [quote from [111]]. There are no such data for RobT carriers; however, human acrocentric chromosomes are known to present a considerable degree of size heteromorphism, and the following naturally occurring variants have been established: 13cenh, 14pstkstk, 14p-, 15centh + , 15pst, 15p-, 21 ps + , 21pss, 22 ps + , 22pstk + , and 22pvar [112–114]. Although the relationship between the presence of heterochromatin variants and infertility is still controversial, a recent study demonstrated a significant increase in chromosome aneuploidy in both sperm and embryos from male carriers of heteromorphic variants [115, 116]. The current prevailing view is that heterochromatic variants around the centromeres of acrocentric chromosomes could alter spindle attachment, chromosome pairing, and cell division, potentially leading to an increased risk of aneuploidy [111].

It seems that variations in the intranuclear sperm architecture that occur in the presence of chromosomal aberrations could be an additional factor in the reproductive failure of RobT carriers, although this possibility remains hypothetical [93, 97, 117–120]. Since we do not know what mechanisms are responsible for the fact that a given RobT does not always affect male fertility, it would be undoubtedly interesting to collect analogous data on fertile RobT carriers, especially in family scenarios. However, we know from our own experience that the potential for such unique sample collection is very limited [121, 122].

## Comment

The view that has remained unchanged for many years is that in reproductive performance, it is vital to link comprehensive research with the appearance of RobTs, since this knowledge may influence genetic counselling for RobT carriers [23, 46]. It is particularly important to obtain results from groups of rare RobT carriers that are several times larger, which would allow the appropriate statistical analysis. At this point, it appears that the poor semen quality, increased aneuploidy frequency, and meiotic segregation patterns of RobT carriers are not exclusively dependent upon the Class 1 and Class 2 classification.

After 30 years of research on sperm chromosomes using the FISH method, we aggregated experimental data on hyperhaploidy levels from 127 carriers and meiotic segregation patterns from 213 carriers of both rare and common RobTs. Furthermore, it is well established that the control results for fertile donors originating from different laboratories differ significantly [49] (this is an intriguing phenomenon, but probably has little in common with spermatogenesis efficiency). It seems that there is not enough data to recommend FISH analyses with automated systems to provide a solution to this problem. Each laboratory in which sperm chromosomes are studied by the FISH method must establish their own aneuploidy control levels [59]. Additionally, several problems related to the three-colour FISH method involving the simultaneous application of centromeric and telomeric probes cannot be excluded. In the results of such analyses, an individual meiotic segregation pattern can be subjected to a minimum error of several percent. Regrettably, no appropriate validation has been performed in an interlaboratory consortium, and certified reference preparations have not been supplied.

At the centre of the problem of infertility analysis lies the small percentage of RobT carriers with normozoospermia but not sperm aneuploidy (the subfertile status of the wives of these carriers must also be strongly taken into account). From the many years of our own observations, the somewhat controversial observation can be made that men and women with slightly diminished fertility occur in couples more often than can be concluded from random monitoring.

Moreover, it should be emphasized that the reported data on sperm chromosomes originate only from the fraction of RobT carriers recorded during medical consultations due to reproductive failure (estimated to be approximately half of RobT carriers). A lack of analogous information regarding fertile RobT carriers is undoubtedly “the missing link” in our understanding of the mystery associated with RobT-based infertility. Expansion of our knowledge is necessary to provide a sound basis for understanding the mechanisms that may explain why carriers of the same RobT show significant differences in spermatogenesis.

While analysing the data, it is necessary to bear in mind that interindividual differences in the behavior of the same chromosomal translocations can result from the fact that the creation of each translocation is an unique event and each must be considered individually. That should be considered irrespectively from methodological differences. Therefore, the combination of different circumstances for each new RobT and the genomic context of the individual in which they arise may have critical influence on the segregation of the trivalents or the production of aneuploidies. This even applies to Class 1 RobTs, which potentially exhibit the same breakpoints within a particular type of RobT. Familial studies would be great to assess the influence of these factors.

Is it still reasonable to assume that the information obtained from sperm FISH analysis constitutes a good prognostic tool for assessing reproductive success [123]? A relatively high frequency (close to 80%) of sperm cells with normal/balanced chromosomes involved in the translocations observed in infertile carriers should suggest a very good prognosis for infertility treatment (IVF/ICSI). However, the fact that most (70%) infertile RobT carriers exhibit an increased frequency of aneuploidy of at least one of the chromosomes that is not involved in a particular translocation may result in a risk of the development of chromosomally imbalanced embryos. Indeed, the previous studies have indicated that an ICE can exist in PGD embryos derived from RobT carriers [124, 125]. It is not known whether this is the reason responsible, but the proportion of abnormal embryos produced by RobT carriers is much higher than the proportion of abnormal sperm cells found there [126]. At present, the prevailing view seems to be that the correlation between the results of sperm chromosome analysis and the proportion of balanced embryos following PGD is rather elusive [34, 105, 127–129]. Interestingly, current data seem to indicate that fertilization by an aneuploid sperm cell giving rise to an imbalanced zygote can be followed by post-zygotic correction through either the loss (in cases of trisomy) or duplication (in cases of monosomy) of the chromosome involved in aneuploidy [130]. This issue is certainly highly controversial, and it is difficult to draw a conclusion about the mechanism of ICE occurrences [124, 129, 130].

What remains to be determined is whether the collection of more data points that are methodologically valid and comparable to those that we have collected so far will be sufficient for the sperm chromosomes of RobT carriers to reveal their secrets.

### Electronic supplementary material

Below is the link to the electronic supplementary material.

Supplementary file1 (DOCX27 kb)

Supplementary file2 (DOCX 66 kb)
