# Supplementary material for: How much, if anything, do we know about sperm chromosomes of Robertsonian translocation carriers?
Source: Cell Mol Life Sci. 2020 Jun 8;77(23):4765–85. doi: 10.1007/s00018-020-03560-5 (PMC7658086; doi:10.1007/s00018-020-03560-5)
Supplement: Supplementary file 1 — Supplementary file1 (DOCX27 kb) [file 18_2020_3560_MOESM1_ESM.docx]

**Supplementary Table 1.** Published data on the individual and mean values of meiotic segregation results in the spermatozoa of rare Robertsonian translocations carriers.

| **Karyotype**  **No. of carriers** | Symmetry | **No. of**  **sperm cells** | **Type of segregation and [%] of segregants** | | | | | **References** |
| --- | --- | --- | --- | --- | --- | --- | --- | --- |
|  |  |  | **alternate** | **adjacent** | **3:0/2n** | | **other** |  |
| **rob(13;15)**  **No. = 17** | DD | ^-^ | 50.9***** | 49.1 | 0.0 | | 0.0 | [43] |
|  |  | ^-^ | 66.8 | 33.2 | 0.0 | | 0.0 |  |
|  |  | ^-^ | 68.9 | 31.1 | 0.0 | | 0.0 |  |
|  |  | 1032 | 71.3 | - | - | | - | [132] |
|  |  | 2304 | 72.8 | 26.2 | 1.0 | | 0.0 | [133] |
|  |  | 1262 | 76.0 | 23.0 | 0.9 | | 0.1 | [134] |
|  |  | 1021 | 76.0 | 23.4 | 0.0 | | 0.6 | [135] |
|  |  | 891 | 77.6 | 20.4 | 1.0 | | 0.0 | [136] |
|  |  | 1281 | 78.1 | 20.2 | 1.6 | | 0.0 | [137] |
|  |  | 882 | 78.3 | 20.6 | 1.0 | | 0.0 | [1348] |
|  |  | 1094 | 78.4 | 20.5 | 0.6 | | 0.5 | [92] |
|  |  | - | 81.3 | 17.6 | 1.1 | | 0.0 | [43] |
|  |  | - | 81.6 | 14.4 | 4.4 | | 0.0 |  |
|  |  | 500 | 81.8 | 16.6 | 1.6 | | 0.0 | [139] |
|  |  | 1109 | 82.7 | 17.0 | 0.0 | | 0.0 | [66] |
|  |  | 67 | 89.6 | 10.4 | 0.0 | | 0.0 | [140] |
|  |  | 2978 | 92.8***** | 6.8 | 0.5 | | 0.0 | [136] |
| **mean value**  **± SD, range:** | | **1202±**765  67-2978 | **76.0±**9.6**^1^**  50.9-92.8 | **22.7±**10.1**^2^**  6.8-49.1 | **0.9±**1.1  0.0-4.4 | | **0.1±**0.3  0.0-0.8 |  |
| **rob(14;15)**  **No. = 10** | DD | - | 68.3***** | 29.8 | 1.9 | | 0.0 | [43] |
|  |  | - | 71.8 | 28.2 | 0.0 | | 0.0 |  |
|  |  | 1000 | 79.9 | 19.00 | 1.1 | | 0.0 | [141,142] |
|  |  | 819 | 81.6 | 15.8 | 0.0 | | 0.0 | [65] |
|  |  | - | 83.3 | 16.7 | 0.0 | | 0.0 | [43] |
|  |  | - | 83.9 | 16.1 | 0.0 | | 0.0 |  |
|  |  | 1548 | 86.4 | 13.8 | 0.0 | | 0.0 | [65] |
|  |  | 4358 | 91.3 | 8.1 | 0.6 | | 0.0 | [136] |
|  |  | 3050 | 92.5 | 7.2 | 0.3 | | 0.0 |  |
|  |  | 1000 | 99.7***** | 0.2 | 0.1 | | 0.0 | [141,142] |
| **mean value**  **± SD, range:** | | **1962±**1432  819-4358 | **83.9±**8.9  68.3-99.7 | **15.5±**8.4  0.2-29.8 | **0.4±**0.7  0.0-1.9 | | **0.0±**0.0  0.0-0.0 |  |
| **rob(21;22)**  **No. = 4** | GG | 350 | 60.0 | 36.0 | 0.0 | | 4.0 | [143] |
|  |  | 1000 | 76.8 | 22.0 | 1.2 | | 0.0 | [144] |
|  |  | 1016 | 85.7 | 13.6 | 0.7 | | 0.0 | [135] |
|  |  | 149 | 96.6 | 3.4 | 0.0 | | 0.0 | [145] |
| **mean value**  **± SD, range:** | | **629**±446  149-1016 | **79.8**±15.5  60.0-97.6 | **18.8**±12.2  3.4-36.0 | **0.5**±0.6  0.0-1.2 | | **1.0**±2.0  0.0-4.0 |  |
| **rob(13;21)**  **No. = 7** | DG | 10000 | 85.6 | 14.3 | | 0.0 | 0.0 | [146] |
|  |  | 3500 | 86.3 | 13.0 | | 0.5 | 0.0 | [147] |
|  |  | 5985 | 86.9 | 11.7 | | 0.8 | 0.0 | [148] |
|  |  | 10223 | 88.4 | 11.1 | | 0.3 | 0.3 | [149] |
|  |  | 10000 | 88.4 | 11.1 | | 0.3 | 0.3 | [150] |
|  |  | 2045 | 91.2 | 8.4 | | 0.2 | 0.2 | [149] |
|  |  | 1000 | 94.4***** | 5.6 | | 0.2 | 0.0 | [66] |
| **mean value**  **± SD, range:** | | **6108±**4013  1000-10223 | **88.7±**3.1**^1^**  85.6-94.4 | **10.7±**2.9**^2^**  5.6-14.3 | | **0.3±**0.3  0.0-0.8 | **0.1±**0.2  0.0-0.3 |  |
| **rob(13;22)**  **No. = 6** | DG | 1000 | 71.9 | 26.4 | | 1.7 | 0.0 | [144] |
|  |  | - | 72.8 | 26.5 | | 0.7 | 0.0 | [43] |
|  |  | 1000 | 81.7 | 13.0 | | 0.7 | 0.0 | [83] |
|  |  | 1124 | 85.5 | 13.9 | | 0.0 | 0.0 | [42] |
|  |  | 7052 | 86.7 | 12.8 | | 0.5 | 0.0 | [3] |
|  |  | 3421 | 86.8 | 9.9 | | 1.3 | 2.0 | [92] |
| **mean value**  **± SD, range:** | | **2719±2**632  1000-7052 | **80.9±**6.9  71.9-86.8 | **17.1±**7.4  9.9-26.5 | | **0.8±**0.6  0.0-1.7 | **0.3±**0.8  0.0-2.0 |  |
| **rob(14;22)**  **No. = 11** | DG | 288 | 69.4***** | - | | - | - | [132] |
|  |  | - | 76.1 | 22.0 | | 1.9 | 0.0 | [43] |
|  |  | 5087 | 78.5 | 20.9 | | 0.5 | 0.0 | [176] |
|  |  | 5237 | 79.0 | 20.4 | | 0.6 | 0.0 |  |
|  |  | 5428 | 79.4 | 20.0 | | 0.6 | 0.0 |  |
|  |  | 3279 | 79.7 | 20.0 | | 0.3 | 0.0 | [138] |
|  |  | 641 | 80.3 | - | | - | - | [132] |
|  |  | 5152 | 80.5 | 18.9 | | 0.5 | 0.0 | [148] |
|  |  | 5330 | 81.8 | 17.6 | | 0.7 | 0.0 | [138] |
|  |  | 258 | 94.2***** | 5.1 | | 0.8 | 0.0 | [151] |
|  |  | 1035 | 94.5***** | - | | - | - | [132] |
|  | **mean value**  **± SD, range:** | **3174±**2342  258-5428 | **81.2±**7.3  69.4-94.5 | **18.1±**5.4  5.1-22.0 | | **0.7±**0.5  0.3-1.9 | **0.0±0.0**  0.0-0.0 |  |
|  |  |  |  |  |  |  |  |  |
| **rob(15;21)**  **No. = 1** | DG | **1002** | **77.7** | - | | - | - | [132] |
| **rob(15;22)**  **No. = 3** | DG | 3000 | 80.9 | 17.7 | | 1.0 | 0.0 | [82] |
|  |  | 3517 | 87.5 | 10.9 | | 1.4 | 0.0 | [147] |
|  |  | 118 | 89.6 | 10.0 | | - | - | [152] |
|  | **mean value**  **± SD, range:** | **2212±**1832  118-3517 | **86.0±**4.5  80.9-89.6 | **12.9±**4.2  10.0-17.7 | | **1.2±**0.3  1.0-1.4 | **0.0±0.0**  0.0-0.0 |  |
| **∑DD,GG,DG: mean value**  **± SD, range:**  **No. = 59** | | **2585**±2657  67-10223 | **79.7**±9.2  60.0-99.7 | **17.7**±8.9  0.2-49.1 | | **0.7**±0.8  0.0-1.9 | **0.2**±0.6  0.0-2.0 |  |

Kruskal-Wallis test adjusted with the Benjamin-Hochberg method was used to compare results between groups and Wilcoxon test was used to compared individual results (p≤0.05 was considered to be statistically significant).

^P1: unpublished result from own patient included to the study (see Supplemental Mat. & Meth.).

Values with significant differences: 1– (p= 0.029); 2– (p= 0.032).

Differences in individual results:  *****Values significantly differ than mean value for the group.

**rob(13;15):** value 50.9% is significantly lower than results over 69.8% (p ≤ 0.042); value 71.3% is significantly lower than 89.6% (p = 0.044) and 92.8% ( p = 0.033); value 92.8% is significantly higher than result under 76.0% (p ≤ 0.038);

**(14;15):** value 68.3% is significantly lower than results over 86.4% (p ≤ 0.046); value 71.8% is significantly lower than results over 91.3% (p ≤ 0.040); value 99.7% is significantly higher than results under 83.3 (p≤0.046);

**rob(21;22):** value 60.0% is significantly lower than 85.7 % (p = 0.023) and 96.6% (p = 0.012); value 76.8% is significantly lower than 96.6% (p = 0.038);

**rob(14;22):** value 69.4% is significantly lower of 94.2% (p= 0.025) and 94.5% (p= 0.020);
