# Supplementary material for: How much, if anything, do we know about sperm chromosomes of Robertsonian translocation carriers?
Source: Cell Mol Life Sci. 2020 Jun 8;77(23):4765–85. doi: 10.1007/s00018-020-03560-5 (PMC7658086; doi:10.1007/s00018-020-03560-5)
Supplement: Supplementary file 2 — Supplementary file2 (DOCX 66 kb) [file 18_2020_3560_MOESM2_ESM.docx]

**Supplementary Table S2A.** Individual aneuploidy results from the spermatozoa of 33 carriers of rare Robertsonian translocations (RobT). The results concern only the hyperhaploidy of chromosomes that are not involved in a particular translocation.

| **RobT** | Semen | **Spermatozoa with hyperhaploidy of chromosomes ( n = 23+1) [%]** | | | | | | | | | | | | | | | | | **2n**  **[%]** | Ref. |
| --- | --- | --- | --- | --- | --- | --- | --- | --- | --- | --- | --- | --- | --- | --- | --- | --- | --- | --- | --- | --- |
|  | param. | **1** | **4** | **6** | **7** | **8** | **9** | **13** | **15** | **16** | **17** | **18** | **20** | **21** | **22** | **XX** | **YY** | **XY** |  |  |
| **rob(13;15)** | OA OAT | -  - | -  - | -  - | **0.34***  **0.54*** | -  - | **0.35***  **0.43*** |  |  | -  - | -  - | 0.12  0.13 | -  - | **0.29***  **0.44*** | **0.29***  **0.24*** | 0.12  0.13 | 0.09  0.10 | 0.10  0.10 | **1.06***  **0.44*** | [134] |
|  | AT | **0.32*** | **0.27*** | - | - | - | **0.44*** |  |  | **0.32*** | - | - | **0.41*** | **0.55*** | - | **0.25*** | **0.32*** | **0.22*** | **0.44*** | [103] |
|  | OAT | **0.78*** | - | - | - | - | - |  |  | **0.57*** | 0.27 | - | - | - | - | **0.14*** | 0.34 | **0.18*** | **0.14*** | [169] |
|  | AT | - | - | - | - | - | - |  |  | - | - | **0.23*** | - | **0.24*** | - | 0.05 | 0.05 | **0.18*** | 0.09 | [102] |
|  | OAT | - | - | - | - | - | - |  |  | - | - | 0.22 | - | 0.20 | - | - | - | - | - | [65] |
|  | OAT | - | - | - | - | - | - |  |  | - | - | 0.19 | - | **1.09*** | - | - | - | - | - | [132] |
| **rob(14;15)**  homozyg. | **N**  OA | -  - | -  - | -  - | -  - | -  - | 0.20  **1.50*** | -  - |  | -  - | -  - | 0.10  0.20 | -  - | -  - | -  - | -  - | -  - | -  - | 0.00  **2.10*** | [144] |
| **rob(14;15)** | OAT  OAT | -  - | -  - | -  - | -  - | -  - | -  - | 0.00  1.09 |  | -  - | -  - | -  - | -  - | 0.00  0.99 | -  - | -  - | -  - | -  - | **6.26***  **1.36*** | [65] |
| **rob(21;22)** | A | - | - | - | - | - | - | - | - | - | - | **0.60*** | - |  |  | - | - | - | **0.50*** | [144] |
| **rob(21;21)** | AT | - | - | - | - | - | - | - | - | - | - | - | - |  | - | 0.49 | 0.40 | 0.49 | 0.17 | [44] |
|  | OAT | **0.38*** | **0.29*** | **0.19*** | c | c | **0.29*** | c | **0.29*** | **0.87*** | c | c | c |  | c | c | c | c | c | [170] |
| **rob(13;21)** | OA  OAT | -  - | -  - | -  - | -  - | -  - | -  - | -  - | -  - | -  - | -  - | 0.03  0.00 | -  - |  | -  - | 0.03  0.44 | 0.03  0.44 | 0.11  0.18 | **-**  **-** | [168] |
|  | AT | - | - | - | - | - | - | - | - | - | - | **0.32*** | - |  | - | - | - | - | **0.57*** | [78] |
|  | OAT | - | - | - | - | - | - | - | - | - | - | 0.08 | - |  | - | - | - | - | **0.42*** | [148] |
|  | OAT | - | - | - | - | - | - | - | - | - | - | 0.03 | - |  | - | - | - | - | 0.05 | [150] |
| **rob(13;22)** | OAT | - | - | - | 0.17 | - | **0.17*** | - | - | - | - | **0.73*** | - | **0.31*** |  | **0.16*** | **0.22*** | **0.33*** | **0.84*** | [134] |
|  | OA | - | - | - | - | - | - | - | 0.14 | - | - | **0.10*** | - | 0.07 |  | - | - | - | **0.53*** | [83] |
|  | A | - | - | - | - | - | **0.50*** | - | - | - | - | **0.50*** | - | - |  | - | - | - | **2.50*** | [144] |
|  | OAT | - | - | 0.12 | - | - | - | - | - | - | - | **-** | - | 0.29 |  | - | - | - | 0.21 | [167] |
|  | OA | - | - | - | - | - | - | - | - | - | - | 0.03 | - | - |  | - | - | - | **0.34*** | [42] |
| **rob(14;22)** | N | 0.24 | 0.27 | - | - | - | 0.23 | 0.26 | 0.28 | 0.18 | - | - | 0.27 | 0.37 |  | 0.05 | 0.07 | 0.21 | 0.33 | [103] |
|  | Azo | - | - | - | - | - | - | - | - | - | - | - | - | 0.30 |  | 0.20 | **0.59*** | **0.49*** | **0.79*** | [151] |
|  | **N**  OAT  OAT | -  -  - | -  -  - | -  -  - | -  -  - | -  -  - | -  -  - | **1.39***  **-**  **-** | -  -  - | -  -  - | -  -  - | **0.38***  **1.29***  **0.78*** | **1.68***  **-**  **-** | -  -  - |  | -  -  - | -  -  - | -  -  - | 0.00  **0.64***  **0.39*** | [132] |
|  | OAT | - | - | - | - | - | - | - | - | - | - | 0.00 | - | - |  | - | - | - | 0.04 | [148] |
|  | AT | - | - | - | - | - | - | - | - | - | - | **0.40*** | - | - |  | - | - | - | **5.70*** | [78] |
| **rob(15;21)** | OAT | - | - | - | - | - | - | **1.30*** | - | - | - | 0.00 | - |  | - | - | - | - | - | [132] |
| **rob(15;22)** | **N** | - | - | - | 0.05 | 0.08 | - | 0.17 | - | - | - | 0.11 | - | 0.42 |  | 0.05 | 0.14 | 0.35 | 0.72 | [82] |
| **Mean ± SD,**  **range** | | **0.43**  ±0.21  0.24-0.78 | **0.28**  ±0.01  0.27-0.29 | **0.16**  ±0.05  0.12-0.19 | **0.28**  ±0.21  0.05-0.54 | **0.08** | **0.46**  **±**0.41  0.20-0.50 | **0.70**±  0.63  0.00-1.39 | **0.24**  ±0.08  0.14-0.29 | **0.49**  ±0.30  0.18-0.87 | **0.27** | **0.28**  ±0.43  0.00-1.29 | **0.79**  **±**0.78  0.27-1.68 | **0.40**  ±0.31  0.00-1.09 | **0.27**  ±0.04  0.24-0.29 | **0.18**  ±0.15  0.03-0.49 | **0.23**  ±0.18  0.03-0.59 | **0.25**  ±0.14  0.10-0.49 | **1.04**  ±1.66  0.00-6.26 |  |

***The r**esults that were described as statistically different from the control values from the same report. Information on the tests used for such an assessment is presented in the original publications; most authors used Chi-square test and results were considered to be statistically significant when p≤0.05.

Mean values calculated for rare RobT carriers were compared by Kruskal-Wallis and Friedman’s rank sum tests (p≤0.05 was considered to be statistically significant). The analysis was performed only for chromosomes 9, 18, 21, XX, YY, XY and 2n for which there were ≥9 results. Mean value of aneuploidy level for chromosome 18 was statistical higher than 2n and 21 was higher than XX.

Supplementary Table S2B. The results of aneuploidy analysis in spermatozoa of 77 carriers of common rob(13;14). The results concern only the hyperhaploidy of chromosomes that are not involved in a particular translocation.

| **RobT** | Semen  paramet. | **Spermatozoa with hyperhaploidy of chromosomes (n = 23+1) [%]** | | | | | | | | | | | **2n [%]** | Ref. |
| --- | --- | --- | --- | --- | --- | --- | --- | --- | --- | --- | --- | --- | --- | --- |
|  |  | **1** | **7** | **8** | **9** | **15** | **18** | **21** | **22** | **XX** | **YY** | **XY** |  |  |
| **rob(13;14)** | OAT  OAT  OAT | -  -  - | 0.13  -  **0.32*** | -  -  - | 0.13  -  0.26* | -  -  - | 0.07  0.00  0.12 | 0.08  0.00  0.00 | **0.16***  0.00  0.00 | **0.30***  **0.32***  0.10 | 0.03  0.11  0.10 | 0.03  0.11  0.10 | 0.24  0.00  0.06 | [134] |
|  | **N N N** O O O  A T  OA OA OAT | -  -  -  -  -  -  -  -  -  -  - | 0.01  0.04  -  -  0.02  -  0.04  0.08  0.03  -  0.02 | 0.00  0.02  0.04  0.04  0.04  0.02  **0.11***  **0.08***  0.03  **0.13***  0.03 | -  -  -  -  -  -  -  -  -  -  - | -  -  -  -  -  -  -  -  -  -  - | 0.03  0.04  0.03  **0.12***  **0.11***  0.05  0.06  **0.10***  0.06  **0.19***  0.03 | 0.05  **0.23***  **0.21***  **0.11***  **0.33***  **0.26***  **0.35***  0.09  **0.37***  **0.34***  **0.21*** | -  -  -  -  -  -  -  -  -  -  - | 0.02  0.05  0.01  0.05  0.01  0.02  0.02  0.02  0.05  0.01  0.02 | 0.01  0.01  0.06  0.02  0.10  **0.13***  **0.23***  0.10  0.07  **0.58***  **0.18*** | 0.05  **0.23***  **0.16***  0.09  **0.49***  0.11  **0.26***  0.05  0.03  **1.16***  0.09 | 0.08  0.22  0.22  **1.28***  **0.45***  0.12  0.22  0.31  **0.55***  **1.63***  0.31 | [82] |
|  | OAT | - | 0.10 | - | 0.08 | - | 0.17 | 0.16 | - | **0.28*** | **0.25*** | **0.30*** | 0.23 | [171] |
|  | AT  OAT | -  - | -  - | -  - | -  - | -  - | **1.77*** | **4.74***  **1.92*** | **2.96***  **2.40*** | **3.35***  **4.42*** | **1.97***  **2.50*** | 0.59  0.96 | **0.88***  **0.96*** | [172] |
|  | A  O  OA | -  -  - | -  -  - | -  -  - | -  -  - | -  -  - | **0.77***  **0.78***  0.30 | **0.80***  0.49  0.40 | -  -  - | 0.20  0.10  0.00 | 0.60  0.10  0.00 | 0.23  0.20  0.10 | **0.83***  **0.88***  0.10 | [160] |
|  | OAT  OAT  OAT | **0.33***  **1.63***  **2.21*** | -  -  - | -  -  - | -  -  - | **0.87***  **-**  **1.87*** | -  -  - | -  -  - | -  -  - | 0.05  **0.21***  **0.20*** | 0.02  0.36  **0.40*** | **0.13***  **0.21***  0.08 | 0.03  0.18  0.03 | [169] |
|  | O | - | - | - | - | - | 0.20 | - | - | 0.06 | 0.40 | 0.06 | 0.30 | [157] |
|  | **N**  AT  OAT  OAT  OAT | -  -  -  -  - | -  -  -  -  - | -  -  -  -  - | -  -  -  -  - | -  -  -  -  - | **2.06***  **1.54***  **2.17***  **3.20***  **2.24*** | -  -  -  -  - | -  -  -  -  - | **1.67***  **4.12***  **3.60***  **5.76***  **2.62*** | **1.67***  0.00  **1.78***  **1.28***  **1.96*** | **0.83***  0.00  0.20  **0.64***  **0.65*** | **0.26***  **1.54***  0.20  **0.64***  **1.09*** | [158] |
|  | OA  OA  OA  OAT  OAT  OAT  OAT | -  -  -  -  -  -  - | -  -  -  -  -  -  - | -  -  -  -  -  -  - | -  -  -  -  -  -  - | -  -  -  -  -  -  - | 0.11  0.12  0.00  0.00  0.05  0.00  0.00 | 0.10  0.07  0.23  0.06  0.31  0.66  0.11 | 0.10  0.07  0.23  0.17  0.18  0.08  0.00 | -  -  -  -  -  -  - | -  -  -  -  -  -  - | -  -  -  -  -  -  - | 0.03  0.10  0.12  0.18  0.42  0.32  0.25 | [153] |
|  | **N**  **N**  A  A  A  OA  OA  OA  OT  OT  OAT  OAT  OAT  OAT | -  -  -  -  -  -  -  -  -  -  -  -  -  - | -  -  -  -  -  -  -  -  -  -  -  -  -  - | -  -  -  -  -  -  -  -  -  -  -  -  -  - | -  -  -  -  -  -  -  -  -  -  -  -  -  - | -  -  -  -  -  -  -  -  -  -  -  -  -  - | **0.38***  0.10  0.18  0.20  0.20  0.00  0.18  0.19  0.00  0.09  **0.47***  0.20  0.10  **0.35*** | 1.28  1.36  0.99  1.38  1.38  -  1.20  1.20  1.50  2.00  -  0.88  0.80  0.89 | -  -  -  -  -  -  -  -  -  -  -  -  -  - | -  -  -  -  -  -  -  -  -  -  -  -  -  - | -  -  -  -  -  -  -  -  -  -  -  -  -  - | -  -  -  -  -  -  -  -  -  -  -  -  -  - | 0.00  0.00  0.00  0.00  0.06  0.00  **0.29***  0.10  0.00  0.00  **0.46***  0.00  **0.35***  0.00 | [132] |
|  | OAT | - | - | - | - | - | 0.00 | 0.10 | - | - | - | - | 0.00 | [65] |
|  | **N**  A  A  A  A  A | **-**  **-**  -  -  - | **-**  **-**  -  -  - | **-**  **-**  -  -  - | **-**  **-**  -  -  - | **-**  **-**  -  -  - | **0.20***  **0.50***  **0.50***  **0.90***  **1.20***  **1.30*** | **-**  **-**  -  -  - | **-**  **-**  -  -  - | **-**  **-**  -  -  - | **-**  **-**  -  -  - | **-**  **-**  -  -  - | **1.20***  **4.70***  **1.00***  **3.90***  **0.90***  **2.20*** | [144] |
|  | N  N  O  T  T  OA  OT  OAT  OAT  - | -  -  -  -  -  -  -  -  -  - | -  -  -  -  -  -  -  -  -  - | -  -  -  -  -  -  -  -  -  - | -  -  -  -  -  -  -  -  -  - | -  -  -  -  -  -  -  -  -  - | **0.15***  **0.15***  0.05  **0.09***  **0.15***  **0.11***  **0.10***  **0.11***  **0.17***  **0.22*** | -  -  -  -  -  -  -  -  -  - | -  -  -  -  -  -  -  -  -  - | -  -  -  -  -  -  -  -  -  - | -  -  -  -  -  -  -  -  -  - | -  -  -  -  -  -  -  -  -  - | **0.69***  **0.52***  0.16  0.17  **0.43***  **0.65***  0.28  **1.68***  **0.47***  0.20 | [83] |
|  | OAT  OAT  OAT  OAT | -  -  -  - | -  -  -  - | -  -  -  - | -  -  -  - | -  -  -  - | 0.00  0.09  0.00  0.00 | -  -  -  - | -  -  -  - | -  -  -  - | -  -  -  - | -  -  -  - | 0.19  0.47  **6.46***  **3.01*** | [148] |
|  | AT  OAT  OAT  OAT  OAT | **-**  **-**  -  -  - | -  -  -  -  - | -  -  -  -  - | -  -  -  -  - | -  -  -  -  - | **0.23***  **0.16**  0.14  0.05  **0.32*** | -  -  -  -  - | -  -  -  -  - | -  -  -  -  - | -  -  -  -  - | -  -  -  -  - | **0.58***  **0.67***  **1.09***  **0.67***  **0.57*** | [78] |
|  | OA | - | - | - | - | - | - | - | - | - | - | - | **0.98*** | [173] |
| **Mean ±SD,**  **range** | | **1.39**±0.96  0.33-2.21 | **0.08**±0.09  0.01-0.32 | **0.05**±0.04  0.00-0.13 | **0.16**±0.09  0.08-0.26 | **1.37**±0.71  0.87-1.87 | **0.36**±0.62  0.00-3.20 | **0.71**±0.86  0.00-4.72 | **0.56**±1.02  0.00-2.96 | **1.04**±1.68  0.00-5.76 | **0.57**±0.74  0.00-2.50 | **0.30**±0.31  0.00-1.16 | **0.68**±1.08  0.00-6.46 |  |

*****The results that were described as statistically different from the control values from the same report. Information on the tests used for such an assessment is presented in the original publications; most authors used Chi-square test and results were considered to be statistically significant when p≤0.05.

Mean values calculated for 77 carriers of rob(13;14) were compared by Kruskal-Wallis and Friedman’s rank sum tests (p≤0.05 was considered to be statistically significant). The analysis was performed only for chromosomes for which there were ≥10 results. Statistical differences between mean values of aneuploidy levels for particular chromosomes are marked with “+” in separate juxtaposition:

| **Chr** | **7** | **8** | **18** | **21** | **22** | **XX** | **YY** | **XY** | **2n** |
| --- | --- | --- | --- | --- | --- | --- | --- | --- | --- |
|  | 7 |  | **+** | **+** | **+** | **+** | **+** |  |  |
|  | | 8 | **+** | **_+_** | **+** |  | **+** | **+** | **+** |
|  |  |  | 18 | **+** |  | **+** |  |  | **+** |
|  |  |  | | 21 |  |  |  | **+** |  |
|  |  |  | | | 22 |  |  |  |  |
|  |  |  | | | | XX |  | **+** |  |
|  |  |  | | | | | YY |  |  |
|  |  |  | | | | | | XY | **+** |
|  |  |  | | | | | | | |

**Supplementary Table S2C.** The results of aneuploidy analysis in spermatozoa samples of 17 carriers of common rob(14;21). The results concern only the hyperhaploidy of chromosomes that are not involved in a particular translocation.

| **RobT** | Semen  paramet. | **Spermatozoa with hyperhaploidy of chromosomes (n = 23+1) [%]** | | | | | | | | | | | **2n [%]** | Ref. |
| --- | --- | --- | --- | --- | --- | --- | --- | --- | --- | --- | --- | --- | --- | --- |
|  |  | **1** | **7** | **8** | **9** | **13** | **15** | **18** | **22** | **XX** | **YY** | **XY** |  |  |
| **rob(14;21)** | OAT | - | 0.12 | - | 0.16 | 0.12 | - | 0.12 | - | 0.18 | 0.08 | 0.16 | 0.16 | [171] |
|  | OAT | - | **0.07*** | 0.07- | - | - | - | **0.27*** | - | 0.02 | 0.08 | **0.28*** | 0.97 | [82] |
|  | OAT | **-** | - | - | - | **0.37*** | - | **0.32*** | - | 0.05 | 0.09 | **0.18*** | **0.32*** | [102] |
|  | OA | **0.67*** | - | - | - | - | - | - | - | 0.00 | 0.00 | 0.52 | **1.42*** | [164] |
|  | N  N  OT  OT  OAT  OAT  OAT | -  -  -  -  -  -  - | -  -  -  -  -  -  - | -  -  -  -  -  -  - | -  -  -  -  -  -  - | **0.89***  **1.49***  **1.96***  **1.00***  -  **1.09***  **1.79*** | -  -  -  -  -  -  - | 0.20  **0.32***  **0.32***  0.00  **0.86***  **0.48***  **0.28*** | -  -  -  -  -  -  - | -  -  -  -  -  -  - | -  -  -  -  -  -  - | -  -  -  -  -  -  - | 0.00  0.00  **0.16***  **0.38***  **0.19***  0.00  1.05* | [132] |
|  | OAT | - | - | - | - | 0.34 | - | 0.32 | - | - | - | - | 0.36 | [65] |
|  | A  A  A | -  -  - | -  -  - | -  -  - | -  -  - | -  -  - | -  -  - | **0.40***  0.10  **0.40*** | -  -  - | -  -  - | -  -  - | -  -  - | **1.10***  0.30  **0.70*** | [144] |
|  | OA  OA | -  - | -  - | -  - | -  - | -  - | -  - | 0.09  0.09 | -  - | -  - | -  - | -  - | 0.00  1.05 | [42] |
| **Mean ±SD,**  **range** | | **0.67** | **0.10**±0.04  0.07-0.12 | **0.07** | **0.16** | **1.00**±0.65  0.12-1.96 | - | **0.29**±0.20  0.00-0.86 | - | **0.06**±0.02  0.00-0.18 | **0.06**±0.00  0.00-0.09 | **0.29**±0.17  0.16-0.52 | **0.50**±0.48  0.00-1.42 |  |

*****The results that were described as statistically different from the control values from the same report. Information on the tests used for such an assessment is presented in the original publications; most authors used Chi-square test and results were considered to be statistically significant when p≤0.05.

Mean values calculated for 17 rob(14;21) carriers were compared by Kruskal-Wallis and Friedman’s rank sum tests (p≤0.05 was considered to be statistically significant). The analysis was performed only for chromosomes 13, 18 and 2n for which there were ≥9 results. Mean value of aneuploidy level for chromosome13 was statistical higher than for 18 and 2n.

**Supplementary Table S2D.** Summary of the results of chromosome aneuploidy analysis in spermatozoa samples of 169 control normozoospermic males (46,XY). No. = number of males in control group.

| **Control** | | **Spermatozoa with hyperhaploidy of chromosomes ( n = 23+1) [%]** | | | | | | | | | | | | | | | | | **2n**  **[%]** | Ref. |
| --- | --- | --- | --- | --- | --- | --- | --- | --- | --- | --- | --- | --- | --- | --- | --- | --- | --- | --- | --- | --- |
| No. | Mean±SD,  range | **1** | **4** | **6** | **7** | **8** | **9** | **13** | **15** | **16** | **17** | **18** | **20** | **21** | **22** | **XX** | **YY** | **XY** |  |  |
| 7 | mean ±SD | - | - | - | 0.13  ±0.07 | - | 0.12±0.08 | - | - | - | - | 0.09±0.05 | - | 0.11**±**0.07 | 0.08**±**0.06 | 0.11**±**0.09 | 0.10±0.05 | 0.08±0.02 | 0.07**±**0.02 | [174,175] |
| 3 | mean, range | 0.19  0.14-0.25 | 0.14  0.09-0.20 | - | - | - | 0.20  0.16-0.25 | 0.23  0.11-0.30 | 0.18  0.11-0.22 | 0.19  0.15-0.24 | - | - | 0.15  0.10-0.19 | 0.29  0.22-0.35 | - | 0.08  0.07-0.09 | 0.08  0.07-0.10 | 0.11  0.09-0.13 | - | [103] |
| 10 | mean | - | - | - | 0.03  0.02-0.03 | 0.05  0.02-0.07 | - | 0.04  0.03-0.06 | - | - | - | 0.04  0.03-0.05 | - | 0.13  0.09-0.21 | - | 0.03  0.02-0.06 | 0.05  0.04-0.08 | 0.09  0.04-0.12 | 0.27  0.22-0.34 | [82] |
| 4 | mean | - | - | - | 0.16 | - | 0.13 | 0.14 | - | - | - | 0.06 | - | 0.15 | - | 0.10 | 0.07 | 0.08 | 0.29 | [171] |
| 7 | mean ±SD | - | - | - | - | - | - | - | - | - | - | 0.13±0.05 | - | 0.53±0.23 | 0.58±0.27 | 0.15±0.05 | 0.31±0.05 | 0.65±0.17 | 0.16±0.08 | [172] |
| 10 | range | - | - | - | - | - | - | 0.05-0.13 | - | - | - | 0.03-0.10 | - | 0.05-0.10 | - | 0.00-  0.05 | 0.00-0.08 | 0.00-0.10 | 0.00-0.05 | [102] |
| 9 | mean,  range | - | - | - | - | - | - | 0.20  0.06-0.47 | - | - | - | 0.06  0.00-0.18 | - | 0.25  0.05-0.80 | - | 0.23  0.05-0.71 | 0.23  0.05-0.71 | 0.20  0.05-0.40 | - | [168] |
| 5 | mean, range | - | - | - | - | - | - | - | - | - | - | 0.08  0.00-0.14 | - | - | - | 0.06-  0.10 | 0.00-0.10 | 0.08  0.00-0.20 | 0.19  0.05-0.50 | [157] |
| 6 | mean | - | - | - | - | - | - | - | 0.09 | - | - | 0.03 | - | 0.07 | 0.04 | - | - | - | 0.19 | [83] |
| 20 | mean | - | - | - | - | - | - | 0.06 | - | - | - | 0.03 | - | 0.07 | - | - | - | 0.19 | 0.22 | [ 42, 173] |
| 15 | mean | - | - | - | - | - | - | - | - | - | - | 0.12 | - | - | - | 0.28 | 0.28 | 0.42 | 0.16 | [158] |
| 10 | mean | - | - | - | - | - | - | 0.25 | - | - | - | 0.12 | - | 0.29 | - | - | - | - | 0.03 | [132] |
| 5 | mean±SD | - | - | - | - | - | - | - | - | - | - | - | - | 0.16±0.08 | - | - | 0.04±0.03 | 0.07±0.05 | 0.27±0.07 | [151] |
| 6 | mean | - | - | - | - | - | - | - | - | - | - | 0.09 | - | 0.37 | 0.11 | - | - | - | 0.24 | [153] |
| 13 | mean | - | - | - | - | - | - | 0.08 | - | - | - | 0.06 | - | 0.07 | - | - | - | - | 0.03 | [65] |
| 6  1 | mean,  range  **-** | -  - | -  - | -  - | -  - | -  - | -  - | -  - | -  0.51 | -  0.30 | -  0.20 | -  0.55 | -  - | -  - | -  - | 0.02  0.01-0.04  - | 0.03  0.01-0.07  - | 0.13  0.09-0.19  - | 0.13  0.09-0.27  - | [169] |
| 1 | - | - | - | - | - | - | - | - | - | - | - | 0.19 | - | 0.33 | - | - | - | - | 0.09 | [160] |
| 3 | mean, range | - | - | - | - | - | - | - | - | - | - | 0.03  0.00-0.30 | - | - | - | - | - | - | 0.03  0.00-0.30 | [148] |
| 7 | mean | - | - | - | - | - | - | - | - | - | - | 0.12 | - | - | - | - | - | - | 0.28 | [78] |
| 4 | mean | - | - | - | - | - | - | - | - | - | - | - | - | - | - | - | - | 0.44 | 0.22 | [44] |
| 5 | mean | - | - | - | - | - | - | - | - | - | - | 0.07 | - | - | - | - | - | - | 0.04 | [150] |
| 3 | mean | - | - | - | - | - | - | - | - | - | - | 0.13 | - | - | - | - | - | - | 0.13 | [144] |
| 9 | mean, range | - | - | 0.13  0.05-0.31 | - | - | - | - | - | - | - | - | - | 0.37  0.20-0.54 | - | - | - | - | 0.27  0.09-0.44 | [167] |
| **169** | **Mean±SD**  **range** | **0.19**  0.14-0.25 | **0.14^1^**  0.09-0.20 | **0.13^1^**  0.05-0.31 | **0.11^1,2,3^**  ±0.07  0.02-0.16 | **0.05**  0.02-0.07 | **0.15^1^**  ±0.04  0.04-0.25 | **0.14^1,3^**  ±0.09  0.03-0.47 | **0.26^1^**  ±0.22  0.09-0.51 | **0.25^2^**  ±0.08  0.09-0.30 | **0.20** | **0.11^1,2,3,4^**  ±0.11  0.00-0.55 | **0.15**  0.10-0.19 | **0.21^3^**  ±0.14  0.04-0.80 | **0.20^4^**  ±0.25  0.00-0.85 | **0.11^1,2,3,4^**  ±0.10  0.00-0.71 | **0.12^3,4^**  ±0.11  0.00-0.71 | **0.21^3^**  ±0.19  0.00-0.65 | **0.16^3^**  ±0.19  0.00-0.50 |  |

Mean values calculated for 169 control males were compared by Kruskal-Wallis and Friedman’s rank sum tests (p≤0.05 was considered to be statistically significant). The analysis was performed only for chromosomes for which there were ≥10 results. Statistical differences between mean values of aneuploidy levels for particular chromosomes are marked with “+” in separate juxtaposition:

| **Chr** | **7** | **8** | **9** | **13** | **15** | **16** | **18** | **21** | **22** | **XX** | **YY** | **XY** | **2n** |
| --- | --- | --- | --- | --- | --- | --- | --- | --- | --- | --- | --- | --- | --- |
|  | **7** |  |  |  | **+** | **+** |  | **+** | **+** |  |  | **+** |  |
|  | | **8** |  |  |  |  |  |  |  |  |  |  |  |
|  |  |  | **9** |  | **+** |  |  |  |  |  |  |  |  |
|  |  |  |  | **13** | **+** |  |  | **+** | **+** |  |  | **+** |  |
|  |  |  |  |  | **15** |  | **+** |  |  |  |  |  | **+** |
|  |  |  |  |  |  | **16** | **+** |  |  | **+** | **+** |  | **+** |
|  |  |  |  |  |  |  | **18** | **+** | **+** |  |  | **+** |  |
|  |  |  |  |  |  |  |  | **21** |  | **+** | **+** | **+** | **+** |
|  |  |  |  |  |  |  |  |  | **22** | **+** | **+** |  | **+** |
|  |  |  |  |  |  |  |  |  |  | **XX** |  | **+** | **+** |
|  |  |  |  |  |  |  |  |  |  |  | **YY** |  | **+** |
|  |  |  |  |  |  |  |  |  |  |  |  | **XY** | **+** |

.

| Chro  mo  so  me | **Robertsonian translocation: Rare** | | | | | | | | | | | | | | | | | | **Control** | |
| --- | --- | --- | --- | --- | --- | --- | --- | --- | --- | --- | --- | --- | --- | --- | --- | --- | --- | --- | --- | --- |
|  | No.  of  re-  sults | **rob(13;15)** 7 carriers | **rob(14;15)**  4 carriers | | **rob(21;22)**  1 carrier | | **rob(21;21)^@^**  2 carriers | | **rob(13;21)**  5 carriers | | **rob(13;22)**  5 carriers | | **rob(14;22)**  7 carriers | | **rob(15;21)**  1 carrier | | **rob(15;22)**  1 carrier | | **46,XY**  169 males | |
|  |  | Mean±SD,  range  % | No. | Mean±SD,  range  % | No. | Mean  % | No. | Mean,  % | No. | Mean±SD,  range  % | No. | Mean±SD,  range  % | No. | Mean±SD,  range  % | No. | Mean,  range  % | No. | Mean,  range  % | No. | Mean±SD,  range  % |
| **1** | 2 | **0.55**±0.33  0.32-0.78 | 0 | - | 0 | - | 1 | **0.38** | 0 | - | 0 | - | 1 | **0.24** | 0 | - | 0 | - | 3 | **0.19**±0.05  0.14-0.25 |
| **4** | 1 | **0.27** | 0 | - | 0 | - | 1 | **0.29** | 0 | - | 0 | - | 1 | **0.27** | 0 | - | 0 | - | 3 | **0.**14±0.05**^A^**  0.09-0.20 |
| **6** | 0 | - | 0 | - | 0 | - | 1 | **0.19** | 0 | - | 1 | **0.12** | 0 | - | 0 | - | 0 | - | 9 | **0.13±**0.01**^A^**  0.05-0.31 |
| **7** | 2 | **0.44**±0.14**^1^**  0.34-0.54 | 0 | - | 0 | - | 0 | - | 0 | - | 1 | **0.17** | 0 | - | 0 | - | 1 | **0.05** | 21 | **0.11±**0.07**^1, A,B,C^**  0.02-0.16 |
| **8** | 0 | - | 0 | - | 0 | - | 0 | - | 0 | - | 0 | - | 0 | - | 0 | - | 1 | **0.08** | 10 | 0.05  0.03-0.07 |
| **9** | 3 | **0.41**±0.05**^2^**  0.35-0.44 | 2 | **0.85**±0.91**^2^**  0.20-1.50 | 0 | - | 1 | **0.29** | 0 | - | 2 | **0.34**±0.23  0.17-0.50 | 1 | **0.23** | 0 | - | 0 | - | 14 | **0.15**±0.04**^2,A^**  0.04-0.25 |
| **13** | 0 |  | 2 | **0.55**±0.77**^3^**  0.00-1.09 | 0 | - | 0 | - | 0 |  | 0 |  | 2 | **0.83**±0.78^3^  0.26-1.39 | 1 | **1.30^3^** | 1 | **0.17** | 79 | **0.14**±0.09**^3,A,C^**  0.03-0.47 |
| **15** | 0 |  | 0 |  | 0 | - | 1 | **0.29** | 0 | - | 1 | **0.14** | 1 | **0.28** | 0 |  | 0 |  | 10 | **0.26**±0.22**^,A^**  0.09-0.51 |
| **16** | 2 | **0.45**±0.18  0.32-0.57 | 0 | - | 0 | - | 1 | **0.87^4^** | 0 | - | 0 | - | 1 | **0.18** | 0 | - | 0 | - | 4 | **0.25**±0.08**^4,B^**  0.09-0.30 |
| **17** | 1 | **0.27** | 0 | - | 0 | - | 0 | - | 0 | - | 0 | - | 0 | - | 0 | - | 0 | - | 1 | **0.20** |
| **18** | 5 | **0.17**±0.05  0.12-0.23 | 2 | **0.15**±0.07  0.10-0.20 | 1 | **0.60^5^** | 0 | - | 5 | **0.10**±0.15  0.00-0.32 | 4 | **0.34**±0.33**^5^**  0.03-0.73 | 5 | **0.57**±0.49**^5^**  0.00-1.29 | 1 | **0.00** | 1 | **0.11** | 142 | **0.11**±0.11**^5,A,B,C,D^**  0.00-0.55 |
| **20** | 1 | **0.41** | 0 | - | 0 | - | 0 | - | 0 | - | 0 | - | 2 | **0.98**±1.00  0.27-1.68 | 0 | - | 0 | - | 3 | **0.15**±0.04  0.10-0.19 |
| **21** | 6 | **0.48**±0.39**^6^**  0.20-1.09 | 2 | **0.50**±0.70**^6^**  0.00-0.99 | 0 |  | 0 |  | 0 |  | 3 | **0.22**±0.13  0.07-0.29 | 2 | **0.34**±0.05  0.30-0.37 | 0 |  | 1 | **0.42** | 120 | **0.21±**0.14**^6,C^**  0.04-0.80 |
| **22** | 2 | **0.27**±0.04  0.24-0.29 | 0 | - | 0 |  | 0 | - | 0 | - | 0 |  | 0 |  | 0 | - | 0 |  | 26 | **0.20**±0.25**^D^**  0.00-0.85 |
| **XX** | 5 | **0.14**±0.07  0.05-0.12 | 0 | - | 0 | - | 1 | **0.40** | 2 | **0.24**±0.29 | 1 | **0.16** | 2 | **0.13**±0.11  0.05-0.20 | 0 | - | 1 | **0.05** | 76 | **0.11**±0.10**^,A,B,C,D^**  0.00-0.71 |
| **YY** | 5 | **0.18**±0.14  0.05-0.32 | 0 | - | 0 | - | 1 | **0.49** | 2 | **0.24**±0.29 | 1 | **0.22** | 2 | **0.33**±0.37**^7^**  0.07-0.59 | 0 | - | 1 | **0.14** | 81 | **0.12**±0.11**^7,A,B,C,D^**  0.00-0.71 |
| **XY** | 5 | **0.16**±0.05  0.10-0.22 | 0 | - | 0 | - | 1 | **0.53** | 2 | **0.15**±0.05 | 1 | **0.33** | 2 | **0.35**±0.18  0.21-0.49 | 0 | - | 1 | **0.35** | 105 | **0.21**±0.19**^C^**  0.00-0.65 |
| **2n** | 5 | **0.43**±0.39**^8^**  0.09-1.06 | 4 | **2.43**±2.70**^8^**  0.00-6.26 | 1 | **0.50** | 1 | **0.17** | 3 | **0.35**±0.27  0.05-0.57 | 5 | **0.88**±0.93**^8^**  0.21-2.50 | 7 | **1.13**±2.04**^8^**  0.00-5.70 | 0 | - | 1 | **0.72^8^** | 157 | **0.16±** 0.19**^8,C^**  0.00-0.50 |

**Suppl. Table 3.** Mean values of carriers individual results of aneuploidy analysis in the spermatozoa samples of different types of rare RobT carriers and control group ( based on details data from Suppl. Tables **S2A** and **S2D**).

^@^Homologous Robertsonian translocation. Kruskal-Wallis and Wilcoxon tests was used to evaluate the results obtained (p≤0.05 was considered to be statistically significant).

Statistical differences in mean values of aneuploidy levels for individual chromosomes between different ROBs and control group:

^1^For chromosome 7 control mean value 0.11 is differ than mean value 0.44 for rob(13;15) (p= 0.000);

^2^For chromosome 9 control mean value 0.15 is differ than mean values 0.85 for rob(14;15) (p=0.00);

^3^For chromosome 13 control mean value 0.14 is differ than mean values 1.30 for rob(15;21), 0.83 for (14;22) and for 0.55 for rob(14;15); (p=0.00),

^4^For chromosome 16 control mean value 0.25 is differ than mean value 0.87 for rob(21;21) (p=0.00);

^5^For chromosome 18 control mean value 0.11 is differ than mean values 0.57 for rob(14;22), 0.34 for rob(13;22) and 0.60 for rob(21;22)(p=0.00);

^6^For chromosome 21 control mean value 0.21 is differ than mean values 0.50 for rob(14;15) and 0.48 for rob(13;15) (p=0.00);

^7^For YY control mean value 0.12 is differ than mean values 0.33 for rob(14;22) (p=0.00);

^8^For 2n control mean value 0.16 is differ than mean values 0.72 for rob(15;22), 1.13 for rob(14;22), 0.88 for rob(13;22), 2.43 for rob(14;15) and 0.43 for rob(13;15) (p=0.00).

Statistical differences between mean values of aneuploidy levels for individual chromosomes in Control group:

**^A^**mean values lower than mean value 0.26 for chromosome 15 (p=0.02);

**^B^**mean values lower than mean value 0.25 for chromosome 16 (p=0.02);

^C^mean values lower than mean value 0.21 for chromosomes 21 and XY (p=0.02);

^D^mean values lower than mean value 0.20 for chromosome 22 (p=0.03).
